# HypDB: A functionally annotated web-based database of the proline hydroxylation proteome

**DOI:** 10.1371/journal.pbio.3001757

**Published:** 2022-08-26

**Authors:** Yao Gong, Gaurav Behera, Luke Erber, Ang Luo, Yue Chen

**Affiliations:** 1 Department of Biochemistry, Molecular Biology and Biophysics, University of Minnesota at Twin Cities, Minneapolis, Minnesota, United States of America; 2 Bioinformatics and Computational Biology Program, University of Minnesota at Twin Cities, Minneapolis, Minnesota, United States of America; Institute for Systems Biology, UNITED STATES

## Abstract

Proline hydroxylation (Hyp) regulates protein structure, stability, and protein–protein interaction. It is widely involved in diverse metabolic and physiological pathways in cells and diseases. To reveal functional features of the Hyp proteome, we integrated various data sources for deep proteome profiling of the Hyp proteome in humans and developed HypDB (https://www.HypDB.site), an annotated database and web server for Hyp proteome. HypDB provides site-specific evidence of modification based on extensive LC-MS analysis and literature mining with 14,413 nonredundant Hyp sites on 5,165 human proteins including 3,383 Class I and 4,335 Class II sites. Annotation analysis revealed significant enrichment of Hyp on key functional domains and tissue-specific distribution of Hyp abundance across 26 types of human organs and fluids and 6 cell lines. The network connectivity analysis further revealed a critical role of Hyp in mediating protein–protein interactions. Moreover, the spectral library generated by HypDB enabled data-independent analysis (DIA) of clinical tissues and the identification of novel Hyp biomarkers in lung cancer and kidney cancer. Taken together, our integrated analysis of human proteome with publicly accessible HypDB revealed functional diversity of Hyp substrates and provides a quantitative data source to characterize Hyp in pathways and diseases.

## Abstract 2

This study presents a comprehensive online database and webserver of the proline hydroxylation proteome, using an exportable spectral library that enables deep profiling and label-free quantification of modification targets in cell lines and tissues.

## 1. Introduction

Proline hydroxylation (Hyp), first discovered in 1902, is an important protein posttranslational modification (PTM) pathway in cellular physiology and metabolism [1–4]. As a simple addition of a hydroxyl group to the imino side chain of proline residue, the modification is found to be evolutionarily conserved from bacteria to humans. In mammalian cells, Hyp is largely mediated through the enzymatic activities of 2 major families of prolyl hydroxylases—collagen prolyl 4-hydroxylases (P4HAs) [5–7] and hypoxia-induced factor (HIF) prolyl hydroxylase domain (PHD) proteins [8–12], while there are no known enzymes capable of removing protein-bound Hyp yet. Since the activity of prolyl hydroxylases depends on the cellular collaboration of multiple co-factors, including oxygen and iron, as well as several metabolites, such as alpha-ketoglutarate, succinate, and ascorbate, the Hyp pathway is an important metabolic-sensing mechanism in the cells and tissues.

The most well-characterized Hyp targets are collagen proteins and HIFα family of transcription factors. Hyp on collagens mediated by P4Hs is critical to maintaining the triple-helical structure of the collagen polymer and enabling the proper protein folding after translation. Indeed, adding an electronegative oxygen on the proline 4R position promotes the *trans*-conformation and stabilizes the secondary structure of collagen [1]. Inhibition of collagen Hyp destabilizes the collagen and prevents its export from the ER, therefore inducing cell stress and death [13–15]. HIFα transcription factors are essential to mediate hypoxia-response in mammalian cells [16–18]. Hyp of HIFα proteins mediated by PHD proteins under normoxia condition is recognized by pVHL in the Cullin 2 E3 ligase complex, which leads to rapid ubiquitination and degradation of HIFα proteins [19,20]. Hypoxia condition inhibits HIFα Hyp and degradation, enabling the transcriptional activation of over 100 hypoxia-responding genes [21–23].

In the past 2 decades, numerous studies driven by advances in mass spectrometry-based proteomics technology have reported the identification and characterization of diverse new Hyp targets and the important roles of the modification in physiological functions [24–29]. Hyp has been well known to affect protein homeostasis and the classic example is the PHD-HIF-pVHL regulatory axis. The similar mechanism also regulates the turnover of diverse key transcriptional, metabolic, and signaling proteins, including β2AR, NDRG3, ACC2, EPOR, G9a, and SFMBT1, etc. [30–34]. In addition to pVHL-mediated protein degradation, Hyp also regulates substrate degradation by affecting its interaction with deubiquitinases. For example, the hydroxylation of Foxo3a promotes substrate degradation by inhibiting the interaction with deubiquinase Usp9x, and hydroxylation of p53 enhances its interaction with deubiquitinases Usp7/Usp10 to prevent its rapid degradation [35,36]. P4H-mediated Hyp has also been known to regulate the stability of diverse substrates including AGO2 and Carabin [37,38]. In addition to protein degradation, Hyp can also affect protein–protein interaction to regulate signaling and transcriptional activities. For example, PKM2 hydroxylation promotes its binding with HIF1A for transcriptional activation, Hyp of AKT enhances the interaction with pVHL to inhibit the kinase activity of AKT, and PHD1-mediated hydroxylation of Rpb1 is necessary for its translocation and phosphorylation [39–42]. More recently, TBK1 hydroxylation was identified and found to induce pVHL and phosphatase binding, which decreases its phosphorylation and enzyme activity, while the loss of pVHL hyperactivates TBK1 and promotes tumor development in clear cell renal cell carcinoma (ccRCC) [27,43].

Despite these advances, there is a lack of an integrated and annotated knowledgebase dedicated for Hyp, which underappreciates the functional diversity and physiological significance of this evolutionarily conserved metabolic-sensing PTM pathway. To fill the knowledge gap, we developed a publicly accessible Hyp database, HypDB (http://www.HypDB.site) (**S1 Fig**). The development of the HypDB provides 3 main features—first, a classification-based algorithm for confident identification of Hyp substrates; second, integrated resources based on exhaustive manual literature mining, large-scale LC-MS analysis, and curated public database; and third, a collection of a large spectral library for LC-MS-based site-specific identification from a variety of cell lines and tissues. Furthermore, stoichiometry-based quantification of Hyp sites allows quantitative comparison of site abundance across various proteins and tissues, and the extensively annotated Hyp proteome enables deep bioinformatic analysis, including network connectivity, structural domain enrichment, and tissue-specific distribution study. The online database system allows the community-driven submission of LC-MS datasets to be included in HypDB annotation and the direct export of precursor and fragmentation with spectral library that enables the development of targeted quantitative proteomics and data-independent analysis workflow. We hope that the HypDB will provide critical insights into the functional diversity and network of the Hyp proteome and aid in further mechanistic studies on the physiological roles of the metabolic-sensing PTM pathway in cells and diseases.

## 2. Results

### 2.1. Database construction and analysis workflow

**Fig 1:**
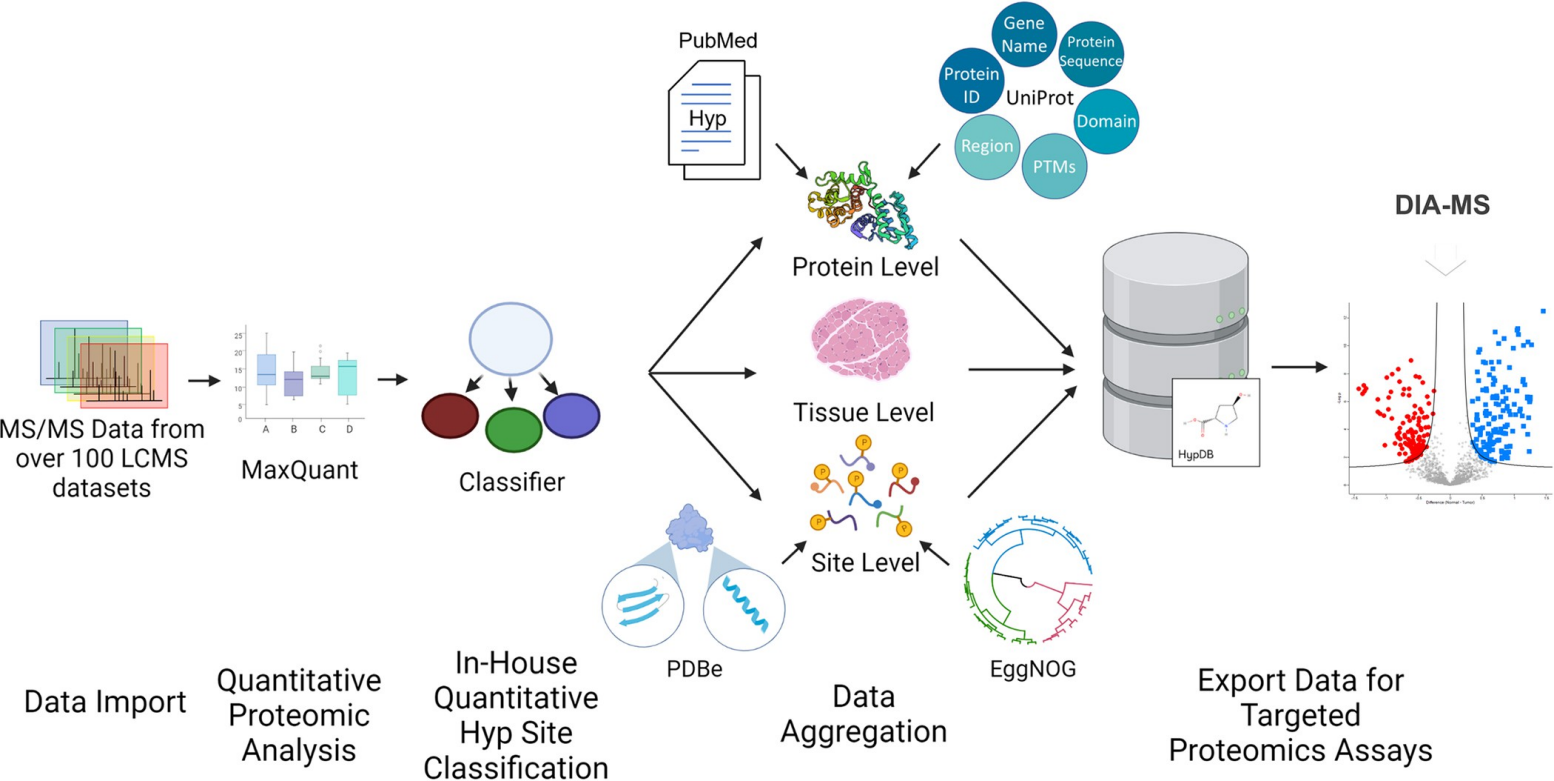
Workflow of establishing HypDB database and webserver. HypDB was constructed through deep proteome profiling analysis of human tissues and cell lines, manual literature mining, and integration with UniProt data source. Classification-based algorithm was applied to extract confident identifications, and site-specific bioinformatic analysis with stoichiometry-based quantification revealed the biochemical pathways involved with human Hyp proteome. MS-based Hyp library further enabled DIA-MS quantification of Hyp proteome in cells and tissues. DIA, data-independent acquisition; Hyp, proline hydroxylation.

To construct a bioinformatic resource for metabolic-sensing Hyp targets, we developed HypDB, a MySQL-based relational database on a public-accessible web server (**Figs 1 and S2**). It was constructed based on 3 main resources to comprehensively annotate human Hyp proteome (**Fig 1**). First, manual curation of literature through PubMed (searching term: “proline hydroxylation” and time limit between 2000 and 2021) was performed by 2 independent curators, which yielded 1,287 research journal articles. Site identification was extracted from each journal article, and its corresponding protein was mapped to UniProt protein ID if possible. Manual curation of the research articles focused on the sites that were biochemically investigated with multiple evidence including mass spectrometry, mutagenesis, western blotting as well as in vitro or in vivo enzymatic assays. Analyzed Hyp site identifications were then matched against the existing data in the database to reduce redundancy. Second, the database included extensive LC-MS-based direct evidence of Hyp site identifications based on the integrated analysis of over 100 LC-MS datasets of various human cell lines and tissues (see Experimental methods). The datasets were either downloaded from publicly accessible server or produced in-house. Each dataset was analyzed through a standardized workflow using MaxQuant search engine, and the Hyp site identifications were filtered and imported into the HypDB with a streamlined bioinformatic analysis pipeline specified in details below. Our collection of MS-based evidence of Hyp identifications from cell lines and tissues likely revealed a significant portion of Hyp sites that can be potentially identified by deep proteomic analysis as evidenced by our observation that the rate of unique Hyp site addition from each dataset decreased significantly despite the increased collection of datasets in the database (**S2B Fig**). Third, the HypDB also integrated Hyp identification annotated in the public UniProt database. For better clarification, the database records indicate whether the site was uniquely reported by the UniProt database or by both UniProt annotation and evidence from large-scale LC-MS analysis.

**Fig 2:**
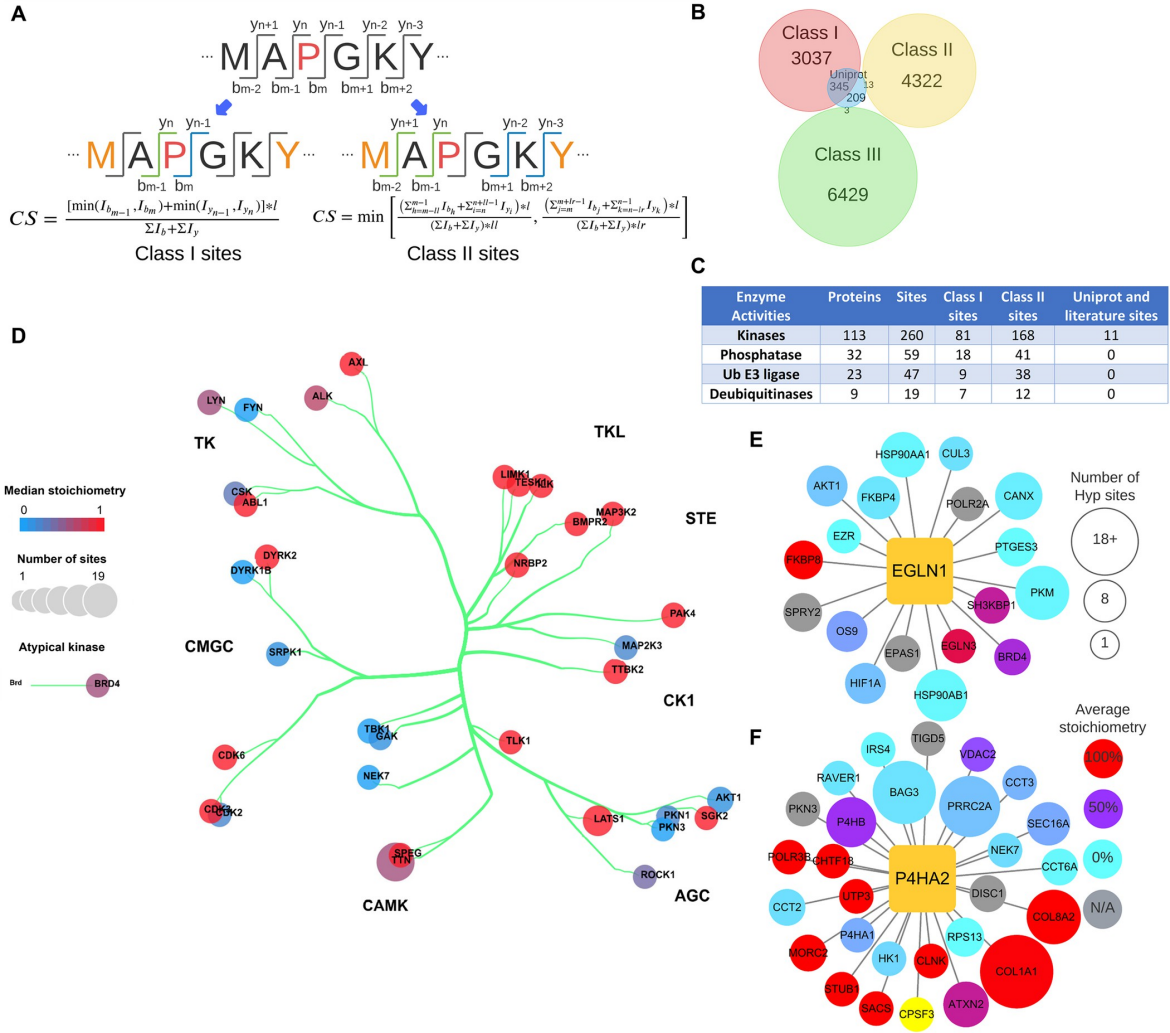
Substrate diversity of the human Hyp proteome. (A) Illustration of classification-based algorithm to identify confident Hyp sites. (B)Venn diagram of Class I, II, III Hyp sites identified from MS analysis and manually curated UniProt sites. (C) PTM regulatory enzymes identified as Hyp substrates. (D) Kinase tree classification showing the distributions of kinases as Hyp substrates in different kinase families, including AGC (named after PKA, PKG, PKC families), CAMK (leaded by calcium/calmodulin-dependent protein kinases), CK1 (cell kinase 1), CMGC (named after CDKs, MAPK, GSK, CLK families), STE (homologs of the yeast STE counterparts), TK (tyrosine kinases), and TKL (tyrosine kinase-like). (E) Hydroxyproline proteins that interact with EGLN1. (F) Hydroxyproline proteins that interact with P4HA2. Refer to Sheet A in S2 Table and Sheet A–G in S3 Table for the underlying data of Fig 2B–2F. Hyp, proline hydroxylation; PTM, posttranslational modification.

We implemented stringent criteria for data importing and classification from LC-MS-based identifications. To import data into the HypDB, LC-MS-based identification of Hyp site from database search analysis was first analyzed by a classification-based algorithm to determine the confidence of Hyp site identification and localization (**Fig 2A**). The classification was performed using the best scored MS/MS spectrum of a Hyp site in each dataset analysis. The algorithm classified Hyp identifications that can be exclusively localized to proline residue based on consecutive b- or y-ions as Class I sites. The algorithm classified the Hyp identifications that cannot be exclusively localized based on MS/MS spectrum analysis but can be distinguished from 5 common types of oxidation artifacts (methionine, tryptophan, tyrosine, histidine, phenylalanine) mainly induced during sample preparation as Class II sites. Other Hyp identifications that were reported by the MaxQuant database search software (with 1% false-discovery rate at the site-level and a minimum Andromeda score of 40) were grouped as Class III sites. We further developed a site-localization score using the relative intensities of key fragment ion to index the level of confidence in site localization with MS/MS spectrum analysis for Class I and Class II sites (**Experimental methods**). Each dataset was analyzed by the classification algorithm separately, and the best classification evidence for each Hyp site was selected and reported on the HypDB website to indicate the confidence of site localization. The classification-based algorithm provides the specificity and reliability required for an accurately annotated database while maintaining all possible identifications as searchable records. And the localization credit score distribution of Class I and Class II sites were shown in **S2C and S2D Fig.**

To evaluate the site-specific prevalence of Hyp, a stoichiometry-based quantification strategy was integrated into the analysis workflow using the previously established principles [27,44]. Briefly, the Hyp stoichiometry was calculated by dividing the summed intensities of the peptides containing the Hyp site identification with the total intensities of the peptides containing the same proline site in the dataset. HypDB recorded all available site-specific Hyp stoichiometry analysis from various cell lines and tissues, which allowed site-specific quantitative analysis of modification abundance across cell and tissue types. And the median stoichiometry of all stoichiometry measurements for any specific site was calculated and reported on the HypDB website.

To further explore the functional association of Hyp proteome, several bioinformatic annotation strategies were integrated into the analysis workflow as a part of the data importing process. These stand-alone workflows include evolutionary conservation analysis, solvent accessibility analysis, and protein–protein interface analysis. Evolutionary conservation analysis compared the conservation of Hyp sites with other proline sites on the same protein and performed a statistical test to determine if the Hyp site is more evolutionarily conserved than non-Hyp sites. Solvent accessibility analysis analyzed the sequence of the substrate protein with DSSP package and calculated the likelihood of solvent accessibility for each Hyp sites. Protein–protein interaction interface analysis extracted the domain interaction residues from the 3DID database based on PDB structure analysis and matched them against the Hyp site in the database to identify the Hyp site that is localized in the interface and more likely to interfere with protein–protein interaction.

All information above was integrated into several tables and linked through foreign keys as the schema in **S2A Fig**. Complete information on all Hyp sites was organized in 2 major tables including a redundant site table (**S1 Table**), which stored all Hyp sites identified in different tissues and cell lines including annotated MS/MS spectra, site-specific abundance and sample source information, and a nonredundant site table (**Sheet A in S2 Table**), which merged the LC-MS-based evidence from different sources at the site-specific level and also integrated with the sites collected from UniProt and manual curation of literatures.

### 2.2. Validation of the Hyp site classification strategy

To validate our classification-based strategy for confidence Hyp site identification, we performed comparative analysis of Hyp site identifications from each class with manually curated UniProt Hyp identifications. Our analysis showed that the Class I sites alone covered over 60% sites annotated in the UniProt, and a combination of Class I and II sites covered about 63% of the UniProt sites, while very few UniProt annotated sites overlapped with the Class III sites (**Fig 2B**), suggesting that our Hyp site localization and classification algorithm allowed the collection of highly confident Hyp identification and significantly improved the reliability of LC-MS-based Hyp site analysis. To further probe the current state of the Hyp proteome, we performed extensive bioinformatic analysis for functional annotation of the Hyp proteome based on more confident Hyp site identifications in HypDB, which excluded Class III only Hyp sites whose LC-MS evidence cannot distinguish them from potential oxidation artifacts.

### 2.3 Mapping human proline hydroxylation proteome

HypDB currently collected 14,413 nonredundant Hyp sites out of 59,436 Hyp site records through large-scale deep proteomics analysis of different tissue, cell lines, manual curation of literatures, and integration with UniProt database. Among 14,413 nonredundant Hyp sites, 3,382 sites were categorized as Class I sites, 4,335 sites were categorized as Class II sites, and 6,432 were categorized as Class III sites (**Fig 2B**). In addition, the database contained 55 sites from literature mining and 209 sites that were integrated from the UniProt database. We applied enrichment analysis with Gene Ontology molecular function annotation and found that Hyp substrates are widely involved in diverse cellular activities, from nucleotide binding and cell adhesion to enzymatic activities such as oxidoreductase and ligases (**S3 Fig and Sheet C in S4 Table**). Excluding Class III Hyp sites, we identified a total of 113 kinases (260 sites), 32 phosphatases (59 sites), 23 E3 ligases (47 sites), and 9 deubiquitinases (19 sites) as Hyp substrates (**Fig 2C and Sheet A–D in S3 Table**). Statistical analysis showed a specific enrichment of kinases in Hyp proteome (*p* = 0.037), suggesting a potentially broad crosstalk between Hyp and kinase signaling pathways (**Fig 2D and Sheet E in S3 Table**). Comparing Hyp substrates with the interactome of prolyl hydroxylases in BioGRID [45], we identified 22 Hyp proteins with 68 sites that were known to interact with EGLN1/PHD2, 17 Hyp proteins with 34 sites that were known to interact with EGLN2/PHD1, 416 Hyp proteins with 861 sites that were known to interact with EGLN3/PHD3, 58 Hyp proteins with 156 sites that were known to interact with P4HA1, 31 Hyp proteins with 296 sites that were known to interact with P4HA2, and 26 Hyp proteins with 66 sites that were known to interact with P4HA3 (**Fig 2E and 2F and Sheet F and G in S3 Table**). The numbers of Class I, Class II Hyp sites, and Hyp proteins that interact with each prolyl hydroxylase were collected in **S4 Fig**.

To determine if Hyp site is more accessible to solvent, we collected 3D structures of proteins from PDBe and UniProt and calculated the relative solvent accessibility (RSA) of each proline residual on proteins with hydroxyproline sites with the DSSP package [46,47]. To examine if there is an RSA difference between Hyp sites and non-Hyp sites on protein with Hyp sites, we performed a 2-tail *t* test and found no significant difference in the distribution of solvent accessibility, suggesting that Hyp does not necessarily target solvent accessible proline residues (**S5A Fig and Sheet C in S2 Table**). To determine if Hyp targets proline sites that are more evolutionarily conserved, we performed evolutionary conservation analysis through extensive sequence alignment of protein orthologs across species based on EggNOG database [48] and statistically compared the conservation of Hyp sites with the conservation of all proline on the same protein. Our data showed that about 49% sites were evolutionarily conserved with statistical significance (*p* < 0.05) (**S5B Fig**). To determine if Hyp could play a potential role in domain–domain interactions, we analyzed data of known domain-based interactions of 3D protein structures from HypDB nonredundant site database. We identified 168 unique Hyp sites that were located at the interface of the interaction. These data suggested potential involvement of Hyp in directly regulating protein–protein interaction. For example, Hyp at position 14 on Superoxide dismutase (SOD1) will form a hydrogen bonding with a neighboring chain Gln16 in a dimeric structure and potentially promote the stabilization of the dimer (**S5C Fig**).

### 2.4. Functional features of proline hydroxylation proteins

**Fig 3:**
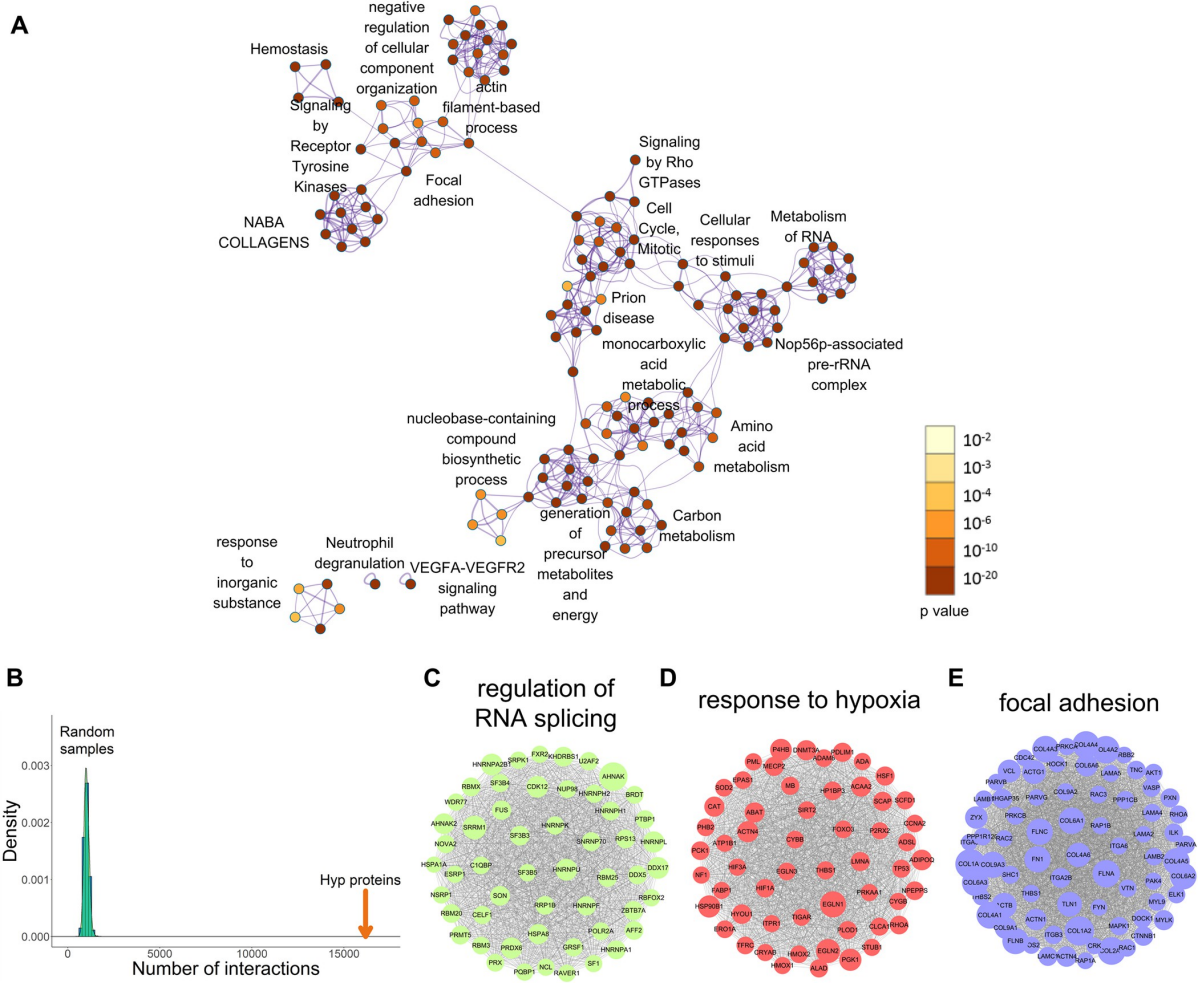
Gene enrichment and connectivity analysis of HypDB. (A) Interaction network of top 20 enriched functional annotation clusters of HypDB proteins. (B) Bootstrapping-based analysis of hydroxyproline protein interactions comparing to a distribution of protein interactions from random samples with the same number of human proteins. (C) Hydroxyproline proteins enriched in the regulation of RNA splicing. (D) Hydroxyproline proteins enriched in the response to hypoxia. (E) Hydroxyproline proteins enriched in focal adhesion. Refer to Sheet A in S4 Table, Sheet D in S2 Table, and Sheet B in S4 Table for the underlying data of Fig 3.

We performed GO enrichment tests and other functional annotations on proteins that contain Class I, II, literature, or UniProt sites (**Fig 3A and Sheet A in S4 Table**). Our analysis revealed that Hyp substrates are highly enriched in metabolic processes such as response to toxic substances (*p* < 10^−26^) and organic cyclic compound catabolic process (*p* < 10^−14^), mRNA splicing (*p* < 10^−26^) and structural functions such as NABA collagens (*p* < 10^−35^), supramolecular fiber organization (*p* < 10^−41^), and cell morphogenesis involved in differentiation (*p* < 10^−18^). To determine if the Hyp proteome prefers to be involved in protein–protein interactions, we extracted a human protein interaction database from STRING with a cutoff score of 0.7, and then, extracted all the interactions containing 2 Hyp proteins based on the STRING database. Based on these data, we performed network connectivity analysis by comparing the number of interactions of Hyp proteins with the distribution of the number of interactions from randomly selected human proteins with 10,000 times of repeats. Our data showed that Hyp substrates are significantly involved in the protein–protein interaction network (*p* < 0.0001) (**Fig 3B and Sheet D in S2 Table**). We further performed protein complex enrichment analysis using manually curated CORUM database, and our analysis showed that Hyp proteome is significantly enriched with many known protein complexes (**S6 Fig and Sheet D in S4 Table**), such as TNF-alpha/NF-kappa B signaling complex 6 (**S7A Fig and Sheet B in S4 Table**), TLE1 corepressor complex (**S7B Fig and Sheet B in S4 Table**), DGCR8 multiprotein complex (**S7C Fig and Sheet B in S4 Table**), Nop56p-associated pre-rRNA complex (**S7D Fig and Sheet B in S4 Table**), and PA700-20S-PA28 complex (**S7E Fig and Sheet B in S4 Table**), suggesting that Hyp targets proteins in multiple pathways that affects signaling and gene expression. Using MCODE clustering analysis, we extracted significantly enriched clusters from Hyp proteome interaction network, and these highly connected clusters of Hyp substrates suggested that Hyp targets important cellular activities including regulation of mRNA splicing, hypoxia response, and focal adhesion (**Fig 3C–3E and Sheet B in S4 Table**).

### 2.5. Structural and motif features of proline hydroxylation sites

**Fig 4:**
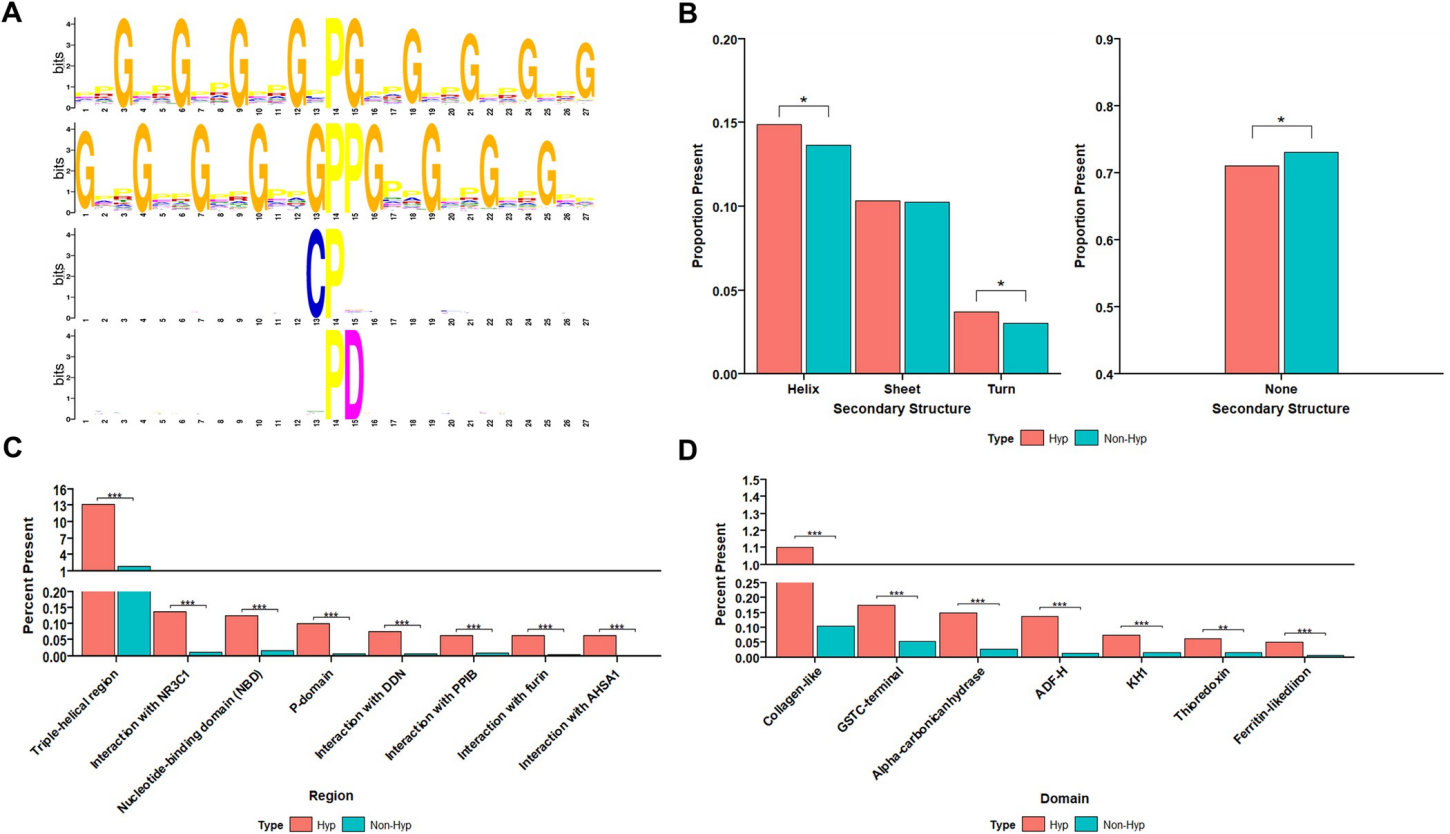
Motif and protein feature analysis of HypDB. (A) Motif enrichment analysis with the flanking sequences of Hyp sites identified PG, GPPG and CP motifs (adj *p* < 10^−6^) and repeated analysis with the flanking sequences of Hyp sites after filtering out PG motif sequences identified PD motif (adj *p* < 10^−6^). (B) Secondary structure enrichment of Hyp sites based on PDB protein structures (*p < 0.05). (C) Functional region enrichment analysis of Hyp sites based on region localizations on proteins in UniProt (***p < 0.001). (D) Functional domain enrichment analysis of Hyp sites based on domain localizations on proteins in UniProt (***p < 0.001, **p < 0.01). Refer to the S5 and S6 Tables for the underlying data of Fig 4.

We analyzed the local sequence context around Hyp sites (excluding Class III sites) using the MoMo software tool [49]. As we expected, Hyp sites with PG motif and GPPG motif were highly enriched (*p* < 10^−10^) which is characteristic for collagen protein families (**Figs 4A and S8A and Sheet A and B in S5 Table)**. In addition to collagen, we identified 33 proteins with similar motif to collagen, and these proteins may be potential substrates of prolyl-4-hydroxylases. Other than the collagen-like motif, we also identified CP motif (*p* < 10^−6^) (**Fig 4A and Sheet C in S5 Table**), and proteins containing CP motifs are highly enriched in focal adhesion (FDR < 0.05). To remove the high background of sites with collagen-like Hyp motifs, we filtered out sites with local sequence contexts in PG motif. Our re-analysis identified that acidic amino acids were enriched at the +1 position to form PD motif (**Fig 4A and Sheet D in S5 Table**). PD motif containing proteins were highly enriched in metabolic pathways (FDR < 0.05). The number and proportion of Hyp sites represented in the HypDB proteome that appeared in the motifs above are shown in **S8B Fig**. As Hyp sites may have crosstalk with other protein, our analysis revealed 2,386 phosphorylation sites and 535 ubiquitination sites that have been identified very close to the Hyp sites (**S8C Fig)**.

To determine the structural features of Hyp sites, we extracted all Hyp proteins with known secondary structures. These proteins contain 2,279 Hyp sites and 27,159 non-Hyp sites on sequences that have experimentally determined PDB structure. We then classified structure features into helix, sheet, turn, and non-structure regions and performed statistical analysis to compare the secondary structure features of Hyp sites and non-Hyp sites. We found that Hyp indeed preferentially targets proline residues that are localized in the helix (*p* < 0.05) and turn secondary structures (*p* < 0.05) (**Fig 4B** left panel). Accordingly, we observed a depletion of Hyp sites outside of a secondary structure feature (**Fig 4B** right panel).

As secondary structures may not fully represent functional structural features, we developed a similar statistical analysis strategy to determine the site-specific enrichment of Hyp sites on functional domains or structural regions. In contrast to the traditional domain enrichment analysis using Pfam or Interpro for protein-level analysis, our strategy enabled site-specific enrichment analysis of domains or regions based on UniProt annotation. Application of this strategy revealed diverse known and novel structural features that were highly enriched with Hyp, such as the triple-helical region, which is characteristic for collagen protein family (**Fig 4C**). In addition to the triple-helical region, our analysis revealed more than 10 functional regions and domains that were highly enriched with Hyp, including p-domain (*p* < 10^−6^), NBD domain (*p* < 10^−6^), thioredoxin domain (*p* < 10^−2^), and ferritin-like domain (*p* < 10^−6^) (**Fig 4C and 4D**). These data revealed previously unexpected role of Hyp targeting functional domains in diverse cellular pathways.

### 2.6. Site-specific stoichiometric quantification of Hyp proteome

**Fig 5:**
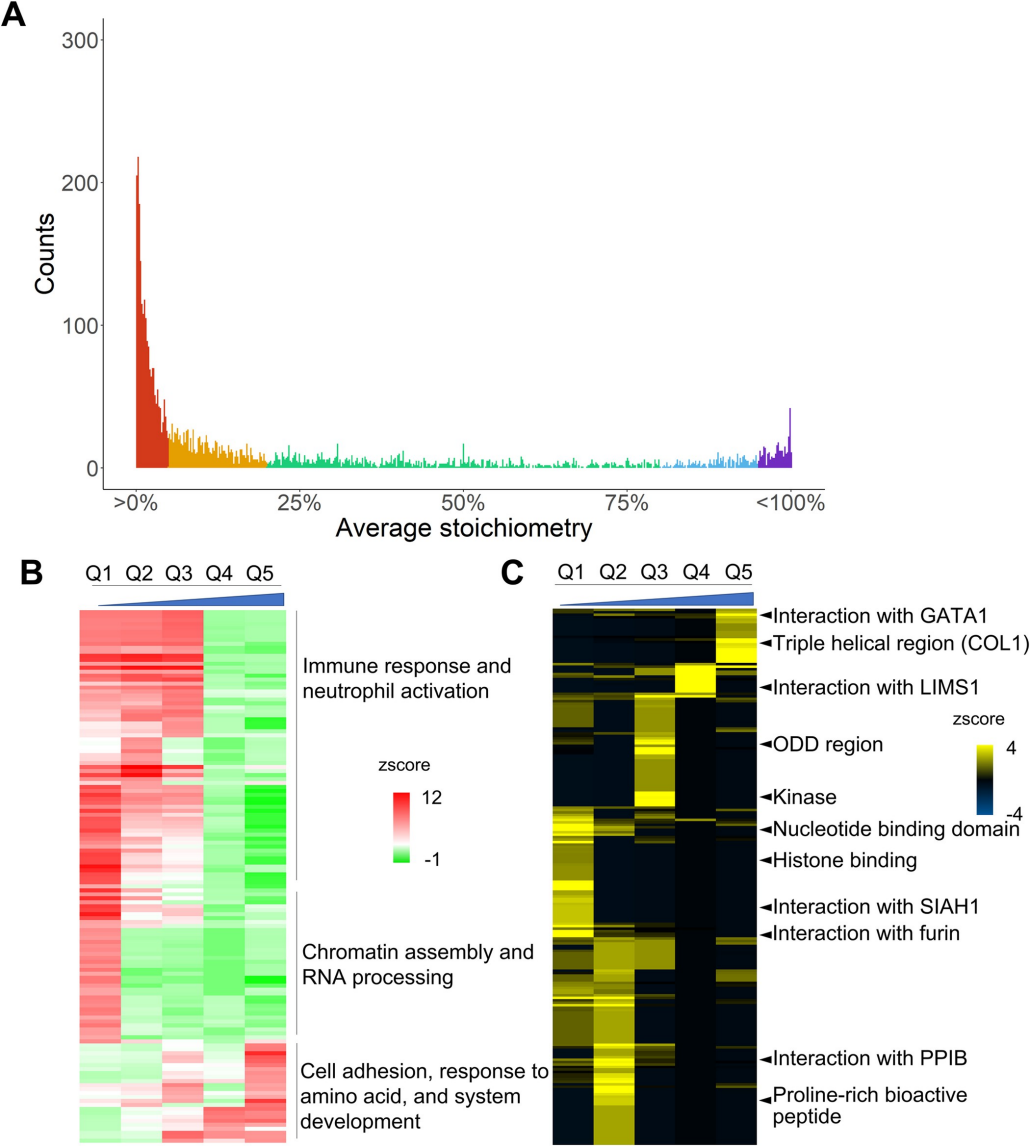
Stoichiometry-based functional enrichment analysis of the Hyp proteome. (A) Stoichiometry distribution of the Hyp sites divided into 5 quantiles—Q1, Q2, Q3, Q4, and Q5, from low to high stoichiometry with 4 cutoffs of 5%, 20%, 80%, and 95% respectively. (B, C) Hierarchical clustering of GO biological processes enrichment of Hyp proteins (B) and functional region enrichment of Hyp sites on proteins in UniProt (C) across the 5 quantiles. Refer to Sheet A–C in S8 Table for the underlying data of Fig 5.

Comparing to relative quantification, stoichiometry analysis measures the prevalence and dynamics of the modification in a physiologically meaningful manner [27,50,51]. Our mass spectrometry-based deep proteome profiling enables site-specific quantification of Hyp stoichiometries across multiple tissues and cell lines. Our data showed that site-specific abundance of Hyp varies widely from below 1% to nearly 100% with an overall median stoichiometry of 7.89% (**Fig 5A and Sheet A in S8 Table**). Indeed, a bulk portion of the Hyp sites have either very low or very high stoichiometries. To investigate the functional differences between sites with different stoichiometry, we divided proteins into 5 quantiles based on average stoichiometry measurement for the same site across all cells and tissues (**Fig 5B and Sheet B in S8 Table**). The 4 cutoffs 5%, 20%, 80%, and 95% were selected so that each quantile contained a similar number of Hyp sites. We then performed GO enrichment and functional annotation on the 5 quantiles respectively and performed hierarchical clustering with correlation coefficient. Our data showed that proteins in immune response and neutrophil activation pathways are enriched with low to medium stoichiometry, and proteins in cell adhesion and system development are enriched with medium to high stoichiometry (**Fig 5B**). We also saw a significant enrichment of proteins involved in chromatin assembly and RNA processing but the stoichiometry of hydroxylation on those proteins seemed to be very low (**Fig 5B**). Combining site-specific functional feature annotation and stoichiometry analysis, we performed stoichiometry-based clustering of Hyp-targeted functional domains. Our data showed that ODD region that is known to regulate hydroxylation-mediated protein degradation of HIFα was enriched with medium stoichiometry, and triple-helical region on collagen, whose hydroxylation is required for its maturation, was enriched with high stoichiometry (**Fig 5C and Sheet C in S8 Table**). Furthermore, our analysis revealed stoichiometry-based enrichment of kinase domains at medium stoichiometry, GATA1 interaction domains at high stoichiometry, nucleotide-binding domains at low to medium stoichiometry, and histone-binding domains at low stoichiometry (**Fig 5C**).

### 2.7. Tissue-specific distribution of Hyp proteome

**Fig 6:**
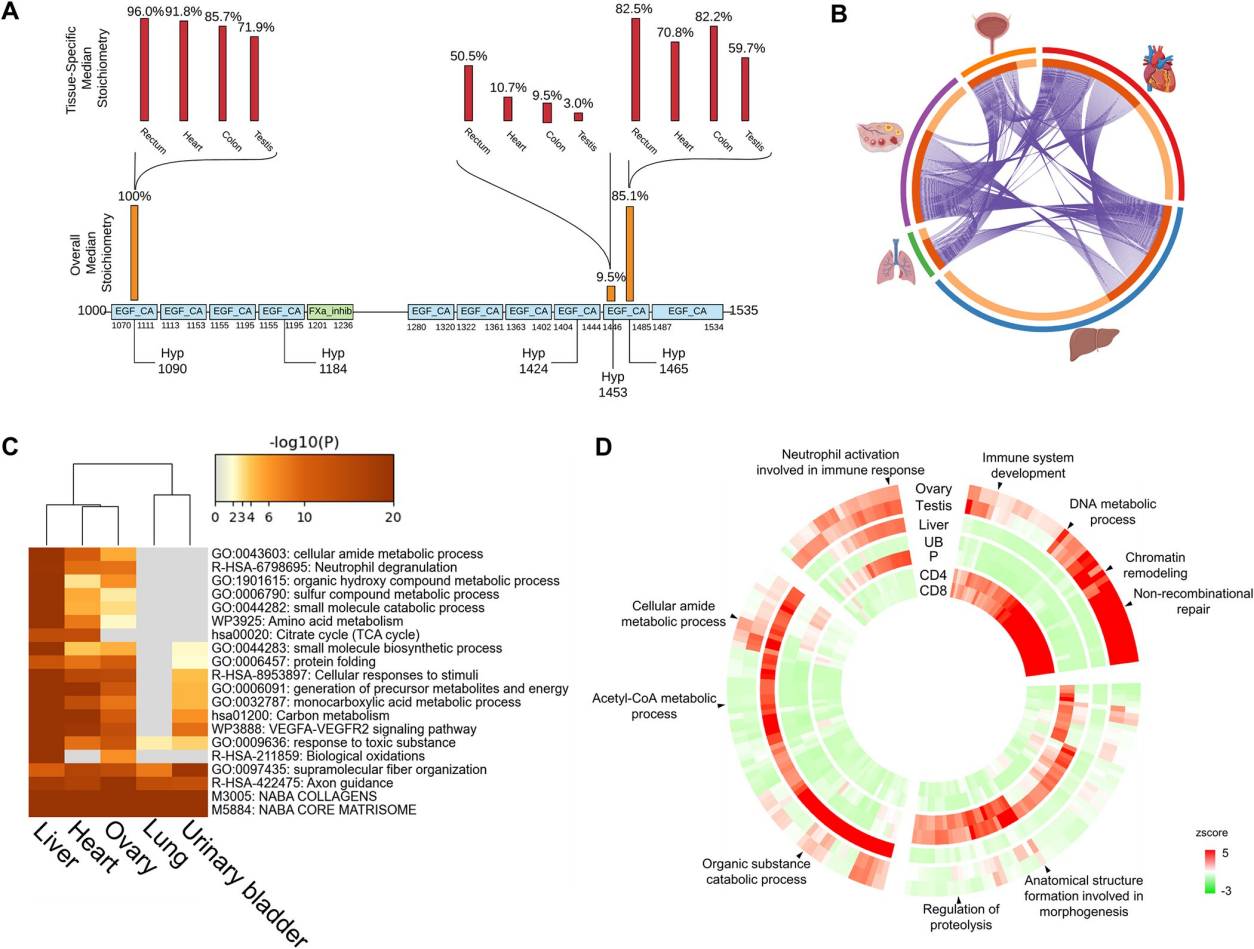
Hyp proteome distributions in different tissues. (A) An example showing varying stoichiometries of Hyp sites across different types of tissue for FBN1 with protein domains labeled in colored boxes. (B) Correlation plot of Hyp proteins in 5 different tissues: heart, liver, lung, ovary, and urinary bladder with the size of arc shows relative number and the purple curved lines showing overlap proteins (C) Heat map of the top 20 enriched functional annotations of the Hyp proteins in 5 tissues. (D) GO biological process enrichment heat map of the Hyp proteins across 7 tissues. Refer to Sheet A in S2 Table, Sheet D-E in S8 Table for the underlying data of Fig 6B–6D. CD4, CD4 T cells; CD8, CD8 T cells; FBN1, Fibrillin-1; Hyp, proline hydroxylation; P, prostate; UB, urinary bladder.

The collection of mass spectrometry-based identification of Hyp proteome enabled cross-tissue comparative analysis (**Sheet A in S8 Table**). Indeed, at individual protein level, we observed a wide distribution of Hyp abundance for the same site and between different sites across different tissue (**Figs 6A and S9**). For example, Fibrillin-1 (FBN1) was identified with 22 Hyp sites of which 17 were Class I or II sites. Hyp1090 on EGF_CA repeat showed consistent high Hyp stoichiometry (71% to 96%) across 4 different tissues (testis, colon, heart, and rectum), while Hyp1453 on another EGF_CA repeat showed varied Hyp stoichiometry (3% to 50.5%) across the same 4 tissues (testis, colon, heart, and rectum) (**Fig 6A**). In another example, 6-phosphogluconate dehydrogenase (PGD) was identified with 8 Hyp sites with half of them belonging to Class I or II sites. Hyp169 on the NAD-binding domain showed relatively low stoichiometries in heart, liver, and ovary (7.6% to 11.6%) but much higher stoichiometries in gut and B cell (21.9% and 75.6%) (**S9B Fig**). We performed pathway enrichment analysis of Hyp and clustering of the enrichment across the tissues. Our data showed that Hyp proteome varied dramatically in terms of pathway and abundance among tissues (**Fig 6B and 6C and Sheet D in S8 Table**). For example, in lung, the Hyp proteome is mainly involved in collagen synthesis and tissue development, and it has relatively low portion of unique Hyp sites, but in liver, the Hyp proteome is heavily involved in diverse metabolic and translational processes with many liver-specific Hyp targets (**Fig 6B and 6C**). Interestingly, clustering analysis showed that tissues sharing similar physiological functions tend to share similar Hyp profiles and are therefore clustered together. Testis and ovary, for example, have similar enrichment of Hyp proteins related to chromosome organization, DNA repair, and other DNA-related metabolic processes (**Fig 6D and Sheet E in S8 Table**). Hyp proteomes in urinary bladder and prostate are co-enriched in regulation of proteolysis and morphogenesis of different tissues. CD4 T cells and CD8 T cells are enriched with Hyp proteins related to chromatin remodeling and immune system development. Liver showed a distinctive enrichment pattern comparing to other tissues, and its Hyp proteome is strongly enriched in various metabolic and catabolic processes. Meanwhile, 4 of these tissues: ovary, testis, liver, and prostate, co-enriched in neutrophil activation involved in immune response (**Fig 6D**).

### 2.8. Data-independent acquisition (DIA) analysis of Hyp targets with HypDB-generated spectral library

DIA has been developed in the past 10 years as a powerful strategy for reliable and efficient quantification of proteins and PTM sites [52–60]. Our extensive collection of the MS-based evidence for human Hyp sites provided an ideal resource to establish a DIA workflow for global, site-specific quantification of Hyp targets in cells and tissues. To this end, our web server has integrated functions for the direct export of annotated MS/MS identification of Hyp sites for selected proteins, cell line, tissue, or at a proteome scale. The Export function provided 2 options—exporting the peptide precursor m/z only or exporting formatted MS/MS spectra. The former option can generate target m/z list that can be used as an inclusion list for targeted quantification of Hyp sites on selected proteins or sites. The latter option can directly generate spectral library used for DIA analysis. Using the Export function, the current HypDB allowed the generation of a comprehensive Hyp spectral library in the NIST Mass Search format (msp) consisting of 6,000 precursor ions, 5,307 peptides, representing 7,717 Class 1 and 2 sites from 3,022 proteins. The webserver was also integrated with the various options for selective exporting. To demonstrate the applicability of our resource in DIA analysis workflow, we analyzed 2 recently published large-scale DIA analysis datasets [55,56]. Both datasets applied DIA analysis to quantify protein dynamics in the multiple replicates of paired normal and tumor samples.

**Fig 7:**
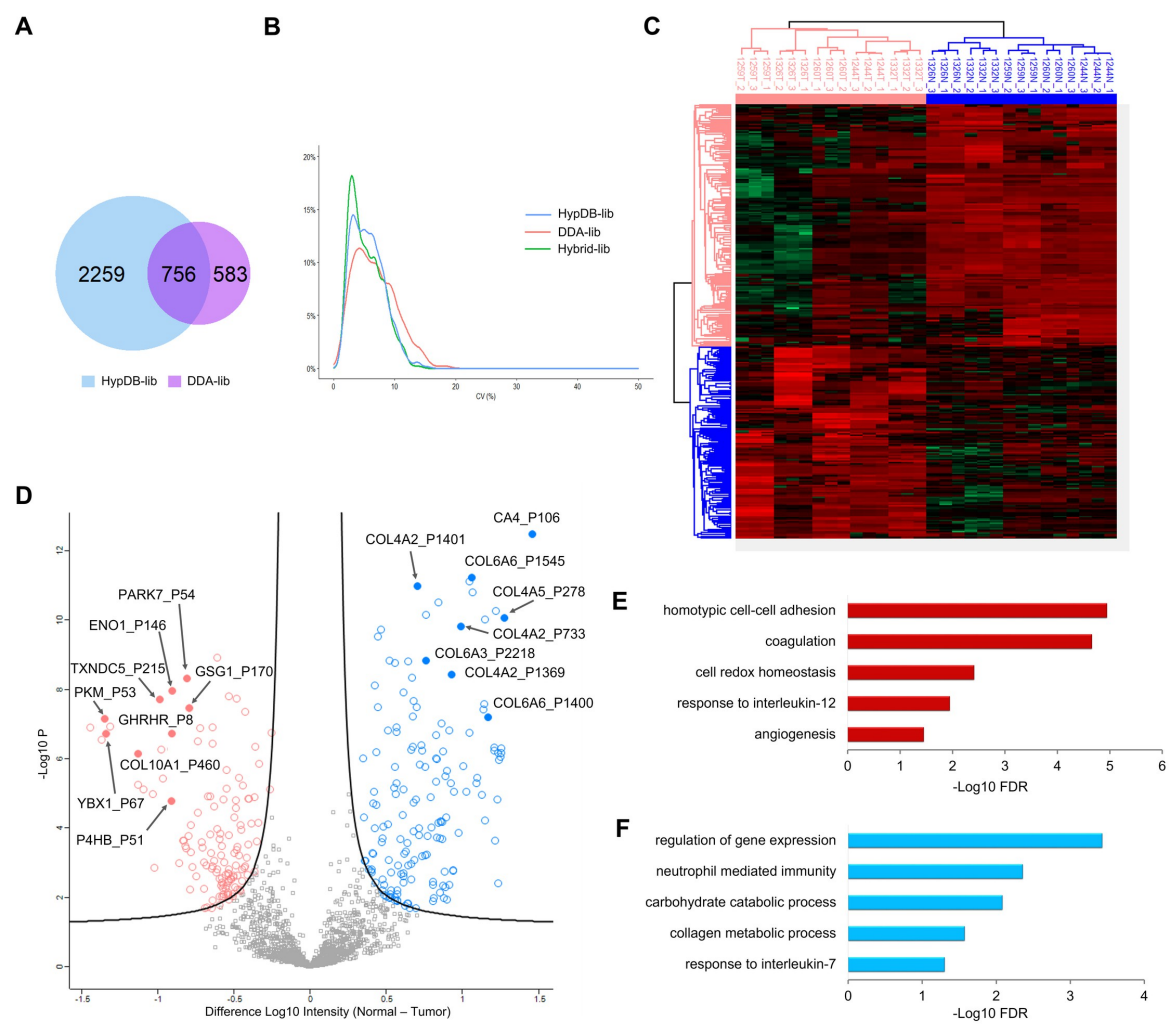
Label-free quantification of the Hyp proteome in lung cancer with DIA analysis. (A) Venn diagram of DIA-based Hyp site identifications using HypDB-generated library and the library generated by the DDA in Kitata and colleagues study. (B) Distribution of %CV for Hyp sites quantified with HypDB-generated library, DDA-generated library, or the hybrid library that combined both sources. (C, D) Hierarchical clustering (C) and volcano plot (D) of significantly up- or down-regulated Hyp sites in normal (blue) and tumor (red) tissues in the DIA analysis. (E, F) Significantly enriched GO biological processes among up-regulated (E) and down-regulated (F) Hyp proteins in tumor with at least 1-fold change after normalizing with protein abundance changes. Refer to Sheet A–E in S9 Table for the underlying data of Fig 7B–F. DDA, data-dependent acquisition; DIA, data-independent acquisition; Hyp, proline hydroxylation.

The study by Kitata and colleagues analyzed global protein profiles of lung cancer with 5 pairs of tumor and normal tissues in triplicate analysis for a total of 30 DIA-based LC-MS runs [55]. As a routine procedure in DIA analysis, we first performed database searching of data-dependent acquisition (DDA) data in the dataset. Then, using the spectral library generated from the DDA data in the same study, we performed DIA analysis of all tumor and normal tissues with replicates. The analysis quantified 1,339 Class 1 and 2 Hyp sites from Kitata and colleagues study (1% FDR). Next, we applied the HypDB-generated spectral library and repeated the DIA analysis. Our result showed that using the HypDB-generated spectral library led to more than double the total number of Hyp sites using a DDA-based spectral library with 3,015 Hyp sites identified while covering more than 83% of the nonredundant Hyp sites identified using the 2 spectral libraries, suggesting that the application of the HypDB-generated spectral library was sufficient to cover majority of the Hyp identifications and significantly increased the sensitivity of Hyp proteome coverage (**Fig 7A**). DIA analysis with a combined library generated by both HypDB and DDA identified 3,651 Hyp sites and 1,249 Hyp proteins (1% FDR). To determine the reproducibility of the quantification, we calculated the distribution of the percentage of coefficient variance (%CV) for DIA analysis of Hyp sites. Our data showed that %CV varied between 2% and 15% with a median value around 5% (**Fig 7B**), similar to the %CV distribution observed in the DIA analysis of proteins and phosphoproteins [55]. Given the high reproducibility of the quantification, we filtered the Hyp sites with a global 1% q-value cutoff (2,283 sites) and performed hierarchical clustering analysis of Hyp sites quantified with normalized intensity in tumor and normal lung tissues (**Fig 7C**). Our data clearly showed that site-specific Hyp quantification was sufficient to cluster and distinguish tumor versus normal tissue. To identify significantly up- or down-regulated Hyp sites in tumor tissues, we performed a 2-sample *t* test and analyzed the data in the volcano plot (**Fig 7D**). The analysis allowed us to identify 142 Hyp sites that were significantly up-regulated and 178 Hyp sites that were significantly down-regulated in tumor tissue (5% permutation-based FDR). The dynamically regulated Hyp sites showed strong characteristics that were distinct between tumor and normal tissue. Interestingly, we observed subtype-dependent Hyp dynamics on collagen proteins. Collagen subtypes IV and VI showed significantly down-regulated Hyp level across multiple sites in tumor samples, while collagen subtype X showed significantly increased Hyp (**Fig 7D**). Since Hyp promotes the structural stability of collagens, such changes likely indicated a significantly increase in stability for collagen X and decrease in stability for collagen IV and VI in lung cancer tissue compared to the normal tissue. Our finding agreed well with a very recent publication indicating a pro-metastatic role of up-regulated collagen X in lung cancer progression [61]. In addition, we also identified significant up-regulation of Hyp on glycolysis enzymes pyruvate kinase (PKM), enolase (ENO1), and autophagy protein Parkin (PARK7) in tumor tissue (**Fig 7D**). P4HB, a member of the collagen prolyl 4-hydroxylase enzyme, also showed significant increase in Hyp (**Fig 7D**), likely due to increased prolyl 4-hydroxylase activity in lung cancer [62].

In another study, Guo and colleagues applied DIA analysis to quantitatively profile kidney cancer proteome and the dataset consisted of an analysis of 18 normal tissues and 18 tumor tissues [56]. Following the same workflow, we first performed DDA analysis and then applied DDA-generated Hyp library to quantify Hyp substrates in tissues. The DDA library-based analysis only quantified 387 Hyp sites from all replicate analysis. Application of the HypDB-generated spectral library increased the number of Hyp site quantifications by more than 5 times, identifying 2,510 sites (**S10A Fig**). Our result confirmed that HypDB-generated library greatly increased the Hyp sequence coverage and analysis sensitivity. DIA analysis with a combined library generated by both HypDB and DDA analysis identified 2,556 Hyp sites and 981 Hyp proteins (1% FDR). To test the reproducibility among replicate tissues, we performed a correlation matrix analysis using the corrplot package in R. Our data showed that quantitative analysis of Hyp substrates allowed efficient clustering and segregation of tumor versus normal tissues (**S10B Fig**). After global q-value filtering and intensity normalization, we analyzed 1,160 Hyp sites across all samples with pair-wise *t* test, and our analysis identified 12 up-regulated sites and 24 down-regulated Hyp sites in tumor (5% permutation-based FDR) (**S10C Fig**).

To understand whether the differential abundance of Hyp sites between the normal and tumor tissues was due to changes in the abundance of corresponding proteins, we compared the log_2_ transformed average site ratios to the log_2_ transformed average protein ratios for both Kitata and colleagues and Guo and colleagues datasets (**S11A and S11B Fig and S9 and S10 Tables)**. We found that more than 82% of the Hyp sites in Kitata and colleagues dataset and at least 37% of the Hyp sites in Guo and colleagues dataset could be quantified with the corresponding protein abundance (**S9 and S10 Tables**). From the correlative analysis between site ratios and protein ratios, we noticed a certain degree of linearity, suggesting the changes in the abundance of some Hyp sites were indeed driven by the changes in the abundance of corresponding proteins (**S11A and S11B Fig**). We also noticed that a significant portion of Hyp site dynamics did not correlate with protein abundance changes. To this end, we calculated 95% confidence interval along the bisector correlation lines that represent equal ratios of Hyp site and protein abundance changes for all Hyp sites with corresponding protein quantification ratios (**S9 and S10 Tables**). Our analysis showed that 78% of the Hyp sites in Kitata and colleagues dataset and 35% of the Hyp sites in Guo and colleagues dataset showed significant deviation in site abundance changes from the corresponding protein abundance changes (**S11A and S11B Fig**). The correlation analysis therefore identified Hyp substrates that showed differential changes in abundances comparing to the corresponding protein abundance changes. We further extracted only the significantly up- or down-regulated Hyp sites based on DIA analysis and compared their dynamics with corresponding protein abundance changes (**S11C–S11F Fig**). Notably, in Kitata and colleagues dataset, the protein abundance of COL1A2 and COL14A1 was similar between tumor and normal tissues, while the abundance of the Hyp sites on each of those proteins were well above or below the 95% confidence interval (**S11C and S11E Fig**). The correlation analysis also confirmed the down-regulation of Hyp abundance on collagen subtypes IV and VI in Kitata and colleagues lung cancer dataset with the protein-level normalization, while showing that the up-regulation of Hyp abundance on collagen subtype X in tumor was due to the up-regulation of the protein abundance (**S11C and S11E Fig**). In Guo and colleagues dataset, significantly changed Hyp sites showed good correlation with corresponding protein dynamics, while the Hyp sites of CRK and TPI1 showed much larger increase or decrease in abundance compared to those of their total proteins, suggesting differential activities of the Hyp pathways for each substrate (**S11D and S11F Fig**).

To reveal the functional significance of up-regulated or down-regulated Hyp substrates in both datasets, we performed functional annotation enrichment analysis with Hyp substrates whose site ratios showed at least 1-fold increase or decrease with protein abundance normalization. Analysis of Kitata and colleagues dataset showed that the biological processes related to homotypic cell–cell adhesion, coagulation, cell redox homeostasis, response to interleukin-12, and angiogenesis were significantly enriched among up-regulated Hyp substrates (**Fig 7E**), while processes related with regulation of gene expression, neutrophil-mediated immunity, carbohydrate catabolism, collagen metabolic process, and response to interleukin-7 were significantly enriched among down-regulated Hyp substrates (**Fig 7F**) (BH corrected FDR < 0.05). The analysis of Guo and colleagues dataset showed that Hyp proteins up-regulated in kidney cancer were strongly enriched in KEGG pathways including ECM-receptor interaction, focal adhesion, glyoxylate/dicarboxylate metabolism, and tryptophan metabolism (**S10D Fig**), while pathways including biosynthesis of amino acids, fructose/mannose metabolism, pathgenic *E*. *coli* infection and PI3K-Akt signaling were significantly enriched among down-regulated Hyp proteins in tumor tissue (**S10E Fig**) (BH corrected FDR < 0.05).

## 3. Conclusions

A grand challenge in functional analysis of PTM pathways is the lack of annotation resources to profile modification substrates and annotate enzyme-target relationships. Hyp is a key oxygen and metabolic-sensing PTM that governs the cellular programs in response to the hypoxia microenvironment and micronutrient stress. Earlier studies of Hyp mainly focused on its role in structural stability and maturation of cytoskeletal proteins such as collagens. In the past several decades, extensive biochemical studies on HIF pathways as well as other new Hyp substrates suggests that Hyp is widely involved in regulating protein–protein interaction, protein stability, signal transduction, metabolism, and gene expression. Growing evidence has also suggested that specific Hyp pathways play critical roles in cancer development, metastasis, heart disease, and diabetes. Systematic categorization and functional annotation of Hyp proteome will provide comprehensive understanding and important physiological insights into Hyp-regulated cellular pathways as well as potential therapeutic strategies targeting metabolic-sensing Hyp pathways in diseases.

To address this need, we developed HypDB, an integrated online portal and publicly accessible server for functional analysis of Hyp substrates and their interaction networks. HypDB collected various data sources for comprehensive coverage of Hyp proteome, including manual curation of published literature, deep proteomics analysis of tissues, and cell lines, as well as integration with annotated UniProt database. The site-localization and classification algorithm enabled efficient extraction of confident Hyp substrate identification from LC-MS analysis. Our identification of highly confident Hyp substrates expanded the current annotation of human Hyp targets in UniProt by over 40-fold. Streamlined data processing and stoichiometry-based Hyp quantification allowed site-specific comparative analysis of Hyp abundance across 26 human organs and fluids as well as 6 human cell lines. We collected 14,413 Hyp sites from various origins, and 86% of the top 500 Hyp sites with the most repeat identifications in various MS datasets were structural proteins, which matched well with one of its most important molecular function.

Bioinformatic analysis of the first draft of human Hyp proteome offer critical insights into the functional and structural diversity of the modification substrates. The analysis not only revealed diverse cellular pathways enriched with Hyp proteins including mRNA processing, metabolism, cell cycle, and signaling, but also demonstrated for the first time that Hyp preferentially targets protein complexes and protein–interaction networks, indicating important roles of Hyp in fine-tuning protein structural features and mediating protein–protein interactions. Indeed, analysis of the expanded Hyp proteome with site-level secondary structure enrichment analysis indicated a significant enrichment of Hyp sites on the alpha-helix, while site-level enrichment analysis of functional domains and regions revealed novel protein domain features that are preferentially targeted by Hyp, such as P-domain, NBD domain, ferritin-like domain, and thioredoxin. These findings suggested potentially important roles for Hyp-mediated regulation of domain stability or activity that are worthy of further biochemical investigation.

MS-based analysis of Hyp proteome allows the stoichiometry-based quantification of Hyp abundance at the site-specific level. By classifying Hyp substrates based on stoichiometry dynamics, we revealed the enrichment of functional domains and activity with very high stoichiometry, indicating that Hyp on those domains may be required for the protein function, which is similar to collagen. In comparison, the oxygen-sensing ODD domain was enriched with median stoichiometry and nucleotide or histone-binding domains were enriched with low stoichiometry. Such difference may suggest differential activities of prolyl hydroxylases targeting various functional domains. Comparative analysis of Hyp stoichiometry across tissues also indicated variations in modification abundance at the site-specific level. Such variation may be attributed to the differential metabolic and gene expression profiles in various tissues.

The collection of MS-based identification of Hyp proteome in HypDB established an annotated spectral library for Hyp-containing peptides that were identified and site localized with high confidence. Such extensive spectral library enabled reliable and sensitive analysis of deep proteomic analysis of human cells and tissues with DIA. Application of the HypDB-generated spectral library in DIA analysis demonstrated excellent data reproducibility, significantly improved the coverage of Hyp proteome in cancer proteome analysis and revealed novel enrichment of Hyp sites that were significantly up-regulated or down-regulated in cancer tissues.

Although the current edition of HypDB (v1.0) is limited to the human proteome, future development of HypDB will include Hyp proteome in other species. Comparative analysis of Hyp targets from diverse species will allow evolutionary conservation analysis of Hyp sites and identify functionally important Hyp targets in protein structure and activity. Further application of the HypDB-generated spectral library in tissue analysis will enable the discovery of novel Hyp targets in disease animal models or patient samples and potentially lead to the development of clinically relevant therapeutic strategies.

## 4. Experimental methods

### 4.1. MS raw data analysis

We collected MS data from the human proteome draft [63], deep proteome analysis of human cell lines [64], PHD interactome analysis [44,65], and Hyp proteome analysis [27] as well as IP-MS analysis of Flag-tagged HIF1A. All MS raw data collected above were searched with MaxQuant (version 1.5.3.12) against the UniProt human database while having carbamidomethyl cystine as fixed modification and protein N-terminal acetylation, methionine oxidation, and Hyp as variable modification. Most of the raw data had trypsin as the digestion enzyme, while a few samples used other digestion enzymes, for example, LysC and GluC, based on the experimental procedure of original projects. Maximum missing cleavage number was set to 2 and the identification threshold was set at 1% false discovery rate for concatenated reversed decoy database search at protein, peptide, and site levels.

### 4.2. Site localization classification and scoring

To filter out low confidence sites, we developed the site localization classification algorithm. Based on the experience that sites are localized more accurately when more ion fragments are found in corresponding MS2 spectra helping to localize the modification mass shift, our algorithm divided sites into 3 classes according to their modification localization confidence: exclusive localized sites in Class I, sites nonexclusive but distinguishable from similar modifications in Class II, and the rest in Class III (**Fig 2A**).

For a site to be classified as Class I site, a pair of b-ions or y-ions separating the proline from other amino acids must be found to localize it exclusively. In this way, a mass shift caused by hydroxylation can only occur on that specific proline. And we gave credits to that ion pair in the scoring function for Class I sites as follows:

$$CS=\frac{[\min\left(I_{b_{m-1}},I_{b_{m}}\right)+\min\left(I_{y_{n-1}},I_{y_{n}}\right)]*l}{\sum I_{b}+\sum I_{y}}$$

where *CS* stands for credit score, *I* stand for intensity of different ion fragments, for example, $I_{b_{m}}$ stands for the intensity of *b_m_*-ion, and *l* stands for peptide length. We gave credit to the pair of b-ions and y-ions that localizes hydroxylation exclusively. The one with lower intensity within the pair will be selected, and we calculate the credit score based on the ratio of their intensities to average ion intensity on the same peptide.

Hydroxylation that cannot be exclusively localized but distinguishable from occurring on other prion-to-oxidize amino acid residuals are classified as Class II because we can infer that hydroxylation occurs on proline in this case. As all ions that separate proline from nearest amino acid may get oxidized easily, we gave credits to all ions that help to separate them in the scoring function for Class II sites as follows:

$$CS=min[\frac{(\sum_{h=m-ll}^{m-1}I_{b_{h}}+\sum_{i=n}^{n+ll-1}I_{y_{i}})*l}{(\sum I_{b}+\sum I_{y})*ll},\frac{(\sum_{j=m}^{m+lr-1}I_{b_{j}}+\sum_{k=n-lr}^{n-1}I_{y_{k}})*l}{(\sum I_{b}+\sum I_{y})*ll}]$$

where *ll* and *lr* for distance between hydroxylated proline and nearest prion-to-oxidation amino acid residual on the left side and right side. Instead of only giving credit to the pair next to the side, for Class II sites, we gave credits to all ions that contributed to separate Hyp with other prion-to-oxidation amino acid residues. We require that Hyp site contains at least 1 fragment ion on both left and right flanking sequences excluding terminal fragment ions. After that, we also calculate the ratio between the average intensity of selected ions and all ions on both sides, and the credit score is determined by the weaker side.

Sites that belong to neither Class I nor Class II are classified as Class III sites. There are chances that Class III sites are Hyp on other positions or other modifications that are identified falsely. Due to their low credibility, we do not score them and only use more confident Hyp identifications, which include Class I, Class II, UniProt, and literature sites for bioinformatic analyses.

### 4.3. Stoichiometry calculation

We calculate the stoichiometry of each hydroxyproline site according to the total peptide intensity and modified peptide intensity. For a specific site, we collect all modified and unmodified peptides that contain this site from MS data. Then, we get stoichiometries by dividing total modified peptide intensity by total peptide intensity. Site stoichiometries in different samples are calculated separately, so there might be multiple original stoichiometries for 1 site in the same tissue or cell line. We take the average stoichiometry for analysis in the following steps in this case.

### 4.4. Statistical enrichment of pathways, functional annotations, domains, and complexes

We use R packages including “GO.db,” “GOstats,” and “org.Hs.eg.db” to perform enrichment analysis including Pfam, Kegg, and Gene Ontology—biological processes, molecular function, and cellular compartment. We collected proteins of Class I, Class II, UniProt, and literature sites from HypDB and performed a hypergeometric test for each term in the annotations above. Enrichment significance is log transformed, and we used Benjamini–Hochberg correction to check the enrichment significance with a cutoff of 0.05.

Meanwhile, we performed enrichment tests by sample and stoichiometry quantiles, respectively. For sample-specific enrichment tests, proteins with hyp sites discovered in different tissues and cell lines are analyzed, respectively. While in the other group, we divide proteins into 5 quantiles according to the average stoichiometry of corresponding sites across all samples. The stoichiometry ranges for 5 quantiles are [0%, 5%), [5%, 20%), [20%, 80%), [80%, 95%), and [95%, 100%). We also perform the log transformation and cluster the samples or quantiles according to the enrichment difference in different terms.

We also used Metascape for functional annotations and visualizations.

### 4.5. Motif enrichment analysis

The protein sequences of the proteins represented in HypDB were downloaded from the UniProt database. In-house Python scripts were written to extract peptides that contained Hyp sites that passed our stringent filtering criteria. These peptides were extended to the length of 27 amino acids and centered around the hydroxylated proline residue. The prealigned peptides were uploaded to the MoMo (version 5.4.1) web application [49]. All protein sequences that were obtained from the UniProt database were set as the background for the analysis. Within the MoMo web application, the motif-x algorithm was selected. The minimum number of occurrences for a motif was set to 20. The sequence logos were generated by the MoMo web application.

### 4.6. Secondary structure analysis

The positions for the secondary structures of the proteins represented in HypDB were downloaded from the UniProt database. In-house Python scripts were developed to determine the number of Hyp sites and non-Hyp sites found in secondary structure features for regions of proteins that have a known PDB structure.

### 4.7. Network connectivity analysis

All Class I, Class II, UniProt, and literature sites in HypDB are collected and transformed into 7,321 ENSP IDs with UniProt. Then, we look for interactions in the String database having both nodes in the ENSP list, and there are 16,176 interactions in total. To test the connectivity significance, we randomly picked 7,321 ENSP IDs from UniProt proteins and counted interactions whose both nodes were included by the randomly selected sample in the String database. The pick and count process are repeated 10,000 times, and these interaction counts from random samples are compared with the corresponding number of sites from HypDB.

We also built a protein–protein interaction network with these hyp proteins. From which we then selected some highly interconnected subnetworks that carry different biological functions with the help of Cytoscape software and the Mcode module.

### 4.8. Solvent accessibility analysis

With information from PDBe and UniProt, we matched hydroxyproline proteins with corresponding pdb ID and protein structures in pdb files. Then, we use R package bio.PDB.DSSP to interpret pdb files that contain structural information and calculate the solvent accessibility of each proline residual in the protein structure using the Sander and Rost accessible surface area (ASA) values. Then, all accessibilities are divided by maximum accessibility of proline to get the relative accessibility number between 0 and 1.

### 4.9. Protein–protein interface analysis

The interacting domain pairs and instances of domain–domain interactions of 3D protein structures were downloaded from 3DID (https://3did.irbbarcelona.org/index.php). In-house Python scripts were developed to analyze the number of Hyp sites interacting with another residue and the number of Hyp sites within 3 residues of an interacting residue.

### 4.10. Evolutionary conservation analysis

Evolutionary conservation analysis of Hyp sites was performed using EggNOG ortholog database (v5.0) and EggNOG-mapper online portal [48]. Briefly, first, using EggNOG-mapper, Hyp proteins were mapped to the corresponding ortholog groups. Next, Hyp sites and non-Hyp proline sites on Hyp proteins were aligned to ortholog sequences using MAFFT algorithm [66]. The number of matches a Hyp site or non-Hyp proline site to a proline for the same positions in ortholog sequences and the total number of sequences in the ortholog group were recorded. Lastly, HyperG test was performed for each Hyp site based on normalized number of matches to proline residues in ortholog sequences for Hyp sites and non-Hyp sites, as well as the total number of any amino acid residues in ortholog sequences for the same position as the Hyp sites or non-Hyp sites.

### 4.11. Development of website and MySQL database

The website serves as a front-end interactive interface of the database. It was developed using HTML, CSS, Javascript, and PHP and works on a Linux-Apache-MySQL-PHP (LAMP) server architecture. The front-end was designed using the Bootstrap framework. Associated protein data are fetched using APIs from several sources. Protein sequences, identifiers, and descriptions are fetched from entries in the UniProtKB/Swiss-Prot knowledgebase [67], protein secondary structure data are fetched from PDBe [68], and domains are fetched from Pfam [69]. The protein sequences are displayed on the website using neXtProt Sequence Viewer (https://github.com/calipho-sib/sequence-viewer). The spectral graphs on the website are visualized using d3.js (https://d3js.org/). The backend of the website utilizes PHP to interface with a MySQL database that contains the data as shown in **S2A Fig**.

### 4.12. Transfection and immunoprecipitation of HIF1A

Transfection and overexpression of Flag-tagged HIF1A was performed following a procedure as previously described [70]. Flag-tagged HIF1A plasmid (Sino Biological) was transfected into 293T cells with polyethylenimine. Cells were treated with 10 μm proteasome inhibitor MG-132 (Apexbio) for 4 hours prior to harvesting. Approximately 24 hours after transfection, cells were washed with cold PBS buffer and lysed in lysis buffer (150 mM NaCl, 50 mM Tris-HCL, 0.5% NP-40, 10% glycerol (pH 7.5), protease inhibitor cocktail (Roche)) on ice for 15 to 20 minutes. Then, the cell lysates were clarified by centrifugation prior to the incubation with anti-FLAG M2 affinity gel (Sigma) for 6 hours at 4°C. After incubation, the M2 gel was washed with wash buffer (cell lysis buffer with 300 mM NaCl) for 3 times and then eluted with 3× Flag peptide (ApexBio). The eluate were mixed with 4× SDS loading buffer and boiled, and then, loaded onto homemade SDS-PAGE gel and stained with Coomassie blue (Thermo Fisher).

### 4.13. In-gel digestion and LC-MS analysis of HIF1A

A large gel piece covering a wide MW range above 100 kDa was cut out and subjected to reduction/alkylation and in-gel digestion with trypsin (Promega) as previously described [51]. Tryptic peptides were desalted with homemade C18 StageTip and resuspended in HPLC Buffer A (0.1% formic acid) before being loaded onto a capillary column (75 μm ID and 20 cm in length) in-house packed with Luna C18 resin (5 μm, 100 Å, Phenomenex). The peptides were separated with a linear gradient of 7% to 35% HPLC Buffer B (0.1% formic acid in 90% acetonitrile) at a flow rate of 200 nl/min on Dionex Ultimate 3000 UPLC and electrosprayed into a high-resolution Orbitrap Lumos mass spectrometer (Thermo Fisher). Peptide precursor ions were acquired in Orbitrap with a resolution of 120,000 at 200 m/z, and peptides were fragmented with Electron Transfer/High Energy Collision Dissociation (EThcd) with calibrated charge-dependent ETD parameters and ETD Supplemental Activation and acquired in Top12 data-dependent mode sort by highest charge state and lowest m/z as priority settings. Raw data were analyzed by Maxquant software following the same procedure and parameter setting as previously published dataset as described above.

### 4.14. Usage of HypDB website

A dedicated website with integrated MySQL database was established to host the HypDB service. The database schema includes 4 tables representing redundant Hyp site identifications, nonredundant Hyp site identifications, interaction interface analysis, evolutionary conservation analysis, and solvent accessibility analysis. Each record in the site identification table is assigned a unique HypDB site ID. The website was designed with the Bootstrap framework (v4.1.3) and features several key functions including a Search bar, Protein information page, Site information page, Database summary, Upload/contribute page, and Download/export page.

The Search bar allows the user to input a UniProt accession number or Gene name of the protein of interest, and the server will use the information to extract and display a ranked list of most similar entries in real time. Clicking on an entry will bring the user to the protein information page where protein identifiers, description, and protein sequence are displayed. All Hyp sites are identified on the protein sequence as well as known acetylation and phosphorylation sites from PhosphoSitePlus database [71] are highlighted by different colors. The list of Hyp sites is further displayed below the sequence in the table that includes the site properties including localization class, localization score, stoichiometry, solvent accessibility, and evolutionary conservation information. Hyp site table is followed by properties of Hyp proteins including protein–protein interaction, secondary structure, functional domains, and domain–domain interactions. Hyp sites identified with MS/MS evidence in the HypDB have a “Details” button displayed for each site in the site table on the protein information page. Clicking on the Details button will bring the user to the peptide information page where the best identified MS/MS spectrum for the site is displayed with annotations of fragment ions.

The Contribute/Upload page allows the community to contribute raw MS/MS identifications to the HypDB through an embedded Google Form. Information regarding the raw data type, location, sample type, database searching parameters as well as user information will be entered into database. Raw data will be downloaded and processed using the same streamlined workflow. The data will pass through the classification and site-localization analysis process and annotated with the bioinformatic workflows as described above. The final data will be deposited into the HypDB to share with the research community.

The Export/Download page allows the community to download the complete dataset deposited in the HypDB including both redundant and nonredundant modification site tables. In addition, the Export function enables users to select a list of proteins, tissues of interests, filter sites based on localization credit class, MS fragmentation type, proteolytic enzyme used in proteomics analysis, as well as specify the precursor ion m/z of Hyp proteins for export to set up targeted quantification method when acquiring data or export the collected spectral libraries of Hyp sites from the selected Hyp proteins to perform database searching with DIA.

### 4.15. Construction of DDA-based spectral libraries

To construct the study-specific DDA-based spectral libraries from Kitata and colleagues and Guo and colleagues studies, a database search of the DDA data from each study was performed by MaxQuant (version 1.5.3.12). The parameters for the search engine were slightly modified from the parameters reported by the authors of each study. The maximum number of cleavages was set to 2 and the threshold for identification was set at 1% FDR. The variable modification of Hyp was included in addition to the variable modifications that the authors of each study reported. The spectral data for Hyp sites were compiled into an msp-formatted spectral library.

### 4.16. DIA data analysis

DIA data were analyzed using DIA-NN (v1.8) [72]. The default workflow for analysis using a spectral library was followed (https://github.com/vdemichev/diann). The DIA data from Kitata and colleagues and Guo and colleagues studies were analyzed separately with DIA-NN. FDR (q-value) for protein groups and Hyp site identification was set at 1.0%. The analysis of the DIA data from each study was performed with spectral library from various sources: HypDB Library, Study-Specific DDA-based Library, and Combined Library generated by both HypDB and Study-Specific DDA Analysis for both Hyp peptide identifications and non-Hyp peptide identifications. DIA-NN further applied global q-value filtering and intensity normalization to generate Hyp site matrix output for Hyp sites that were confidently quantified across all samples. Python scripts developed in-house to process the output from DIA-NN to be Hyp site nonredundant. The matrix output from each study with nonredundant Hyp site quantification was used for clustering, annotation enrichment analysis, and visualization using the Perseus software platform [73]. Missing values were imputed using a normal distribution, and the data were hierarchically clustered. The processed site-nonredundant Hyp intensity data from DIA-NN was also analyzed and visualized using R. Missing values were imputed using the k-nearest neighbor (KNN) method in the NAguideR tool [74].

## Supporting information

S1 Fig Screen shots of the HypDB web portal with front page (top), protein-level view (bottom left), and peptide-level view (bottom right).
(TIF)

S2 Fig The schema of the MySQL database of HypDB and distribution of site properties. (TIF)

S3 Fig Statistical enrichment analysis of Gene Ontology Molecular Function annotation of the Hyp proteome. (TIF)

S4 Fig The total numbers of Hyp sites and corresponding proteins that are known to interact with prolyl hydroxylase. (TIF)

S5 Fig Solvent accessibility, evolutionary conservation, and protein–protein interaction analysis of the Hyp sites. (TIF)

S6 Fig Enrichment of the CORUM protein complexes among the Hyp proteins. (TIF)

S7 Fig Illustrations of Hyp protein networks that are enriched with various CORUM complexes. (TIF)

S8 Fig Flanking sequence motif and neighboring protein modifications of Hyp sites. (TIF)

S9 Fig Examples of Hyp substrate proteins with site-specific Hyp stoichiometries in different tissues. (TIF)

S10 Fig DIA analysis of Guo and colleagues study of kidney cancer revealed differentially regulated Hyp substrates in tumor. (TIF)

S11 Fig Dynamics of the Hyp sites in correlation with corresponding protein abundance changes between normal and tumor tissue. (TIF)

S1 Table The list of redundant Hyp sites collected in HypDB. (XLSX)

S2 Table The list of nonredundant Hyp sites collected in HypDB and its related statistics. (XLSX)

S3 Table Hydroxyproline proteins that act as enzyme that catalyze other PTMs and interact with proline hydroxylases. (XLSX)

S4 Table Annotation enrichment of high confidence proteins in HypDB. (XLSX)

S5 Table Hyp sites identified with PG, GPPG, CP, and PD motifs in HypDB. (XLSX)

S6 Table Motif and protein feature analysis of Hyp sites in HypDB. (XLSX)

S7 Table Overlapping of Hyp sites with other PTMs sites in the neighboring positions of +/− 3 amino acids. (XLSX)

S8 Table Site-specific stoichiometry distribution in tissues and cell lines and its related enrichment results. (XLSX)

S9 Table Hyp identification and quantification with the correlation to the corresponding protein abundance changes from the DIA analysis of Kitata and colleagues study of lung cancer tissues. (XLSX)

S10 Table Hyp identification and quantification with the correlation to the corresponding protein abundance changes from the DIA analysis of Guo and colleagues study of kidney cancer tissues. (XLSX)

## Abbreviations:

ASA: accessible surface area
ccRCC: clear cell renal cell carcinoma
DDA: data-dependent acquisition
DIA: data-independent acquisition
HIF: hypoxia-induced factor
Hyp: proline hydroxylation
KNN: k-nearest neighbor
PGD: 6-phosphogluconate dehydrogenase
P4HA: prolyl 4-hydroxylase
PHD: prolyl hydroxylase domain
PTM: posttranslational modification
RSA: relative solvent accessibility
