# Supplementary figures and images for: HypDB: A functionally annotated web-based database of the proline hydroxylation proteome
Source: PLoS Biol. 2022 Aug 26;20(8):e3001757. doi: 10.1371/journal.pbio.3001757 (PMC9455854; doi:10.1371/journal.pbio.3001757)

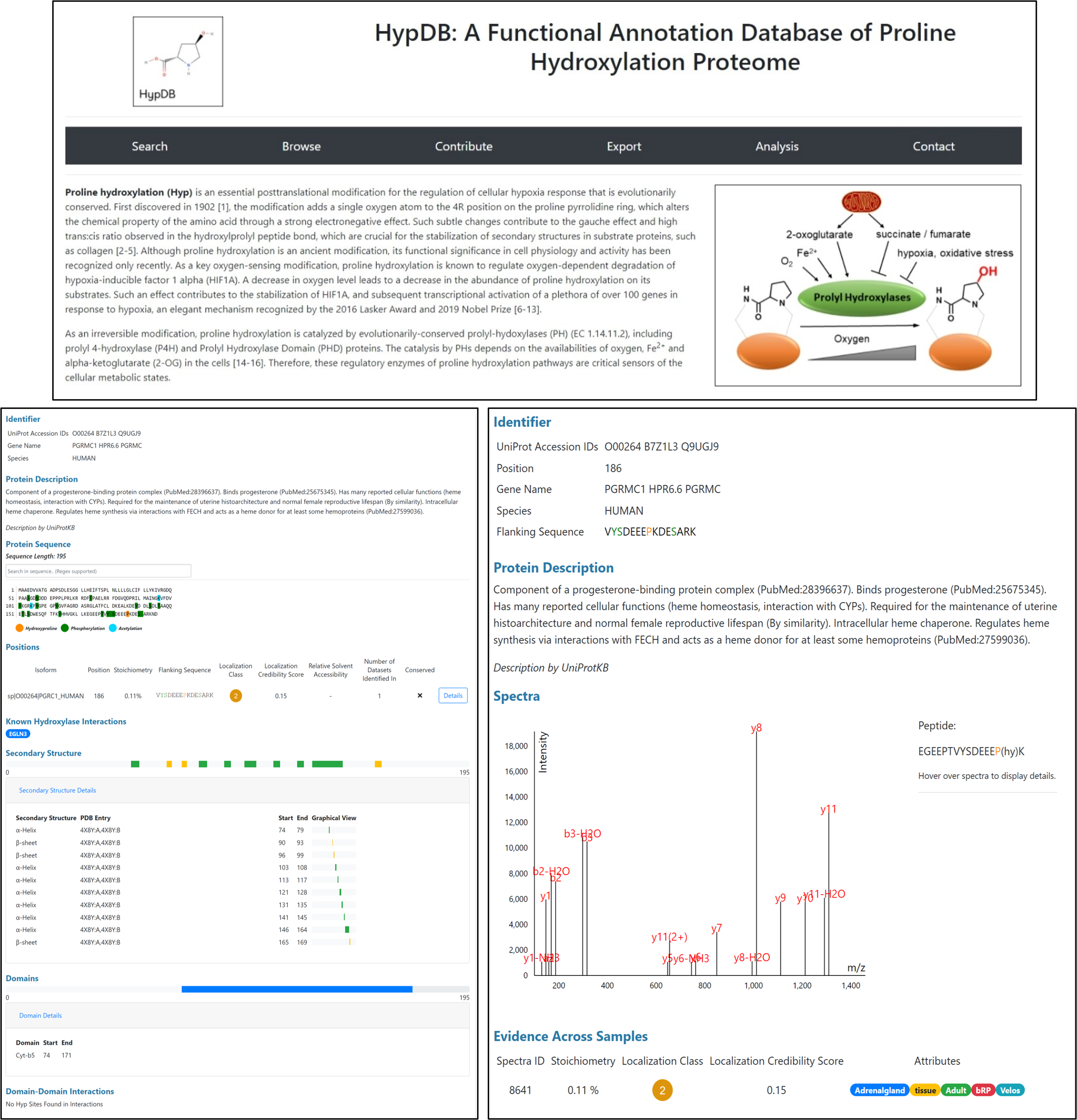

Supplement: S1 Fig — Screen shots of the HypDB web portal with front page (top), protein-level view (bottom left), and peptide-level view (bottom right). (TIF) [file pbio.3001757.s001.tif]

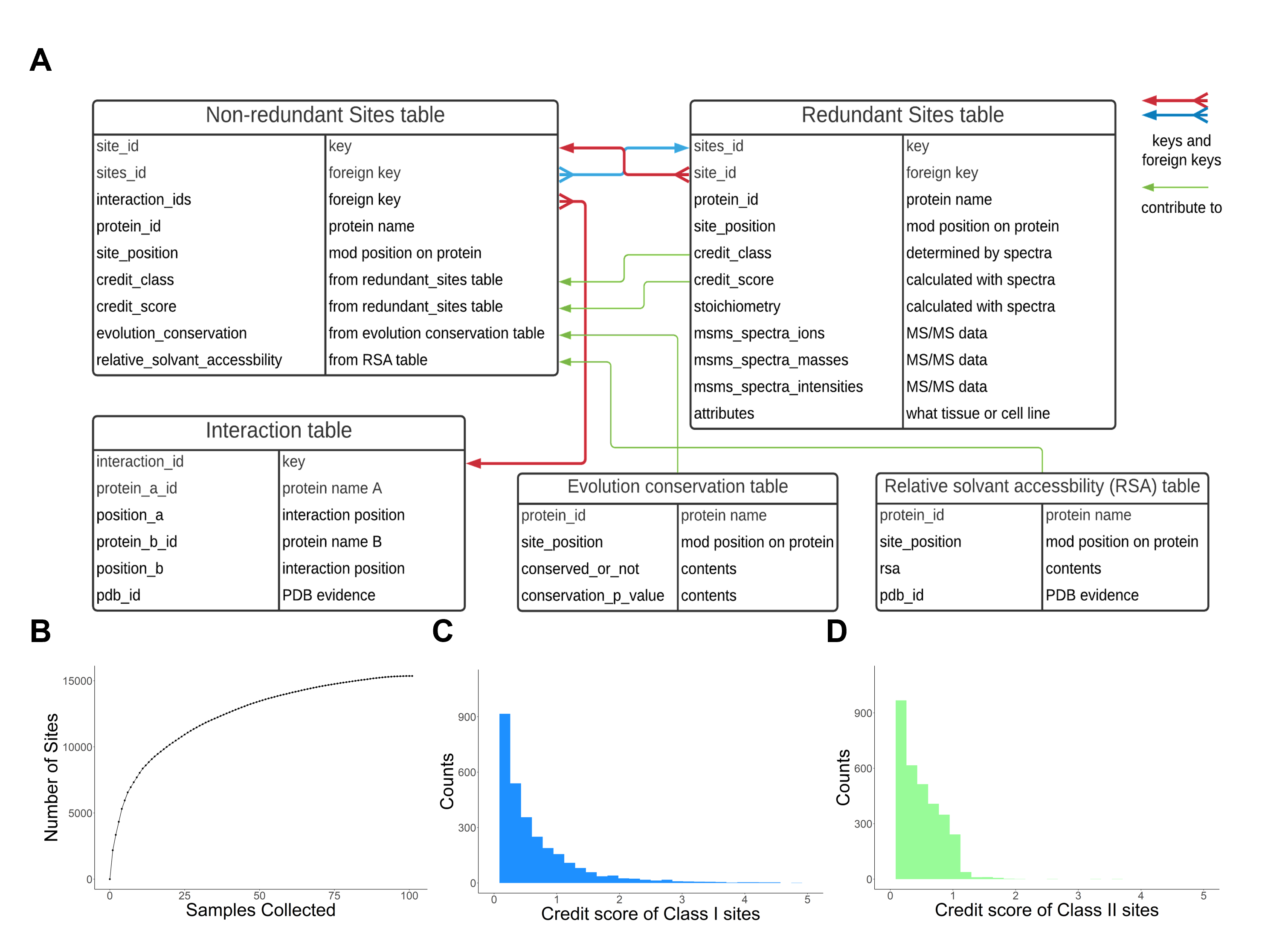

Supplement: S2 Fig — (TIF) [file pbio.3001757.s002.tif]

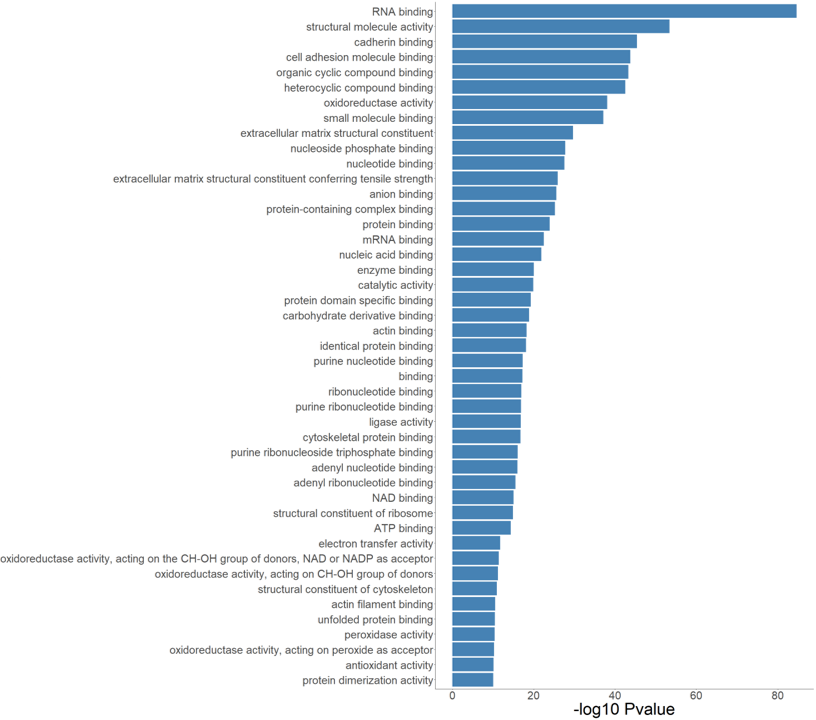

Supplement: S3 Fig — (TIF) [file pbio.3001757.s003.tif]

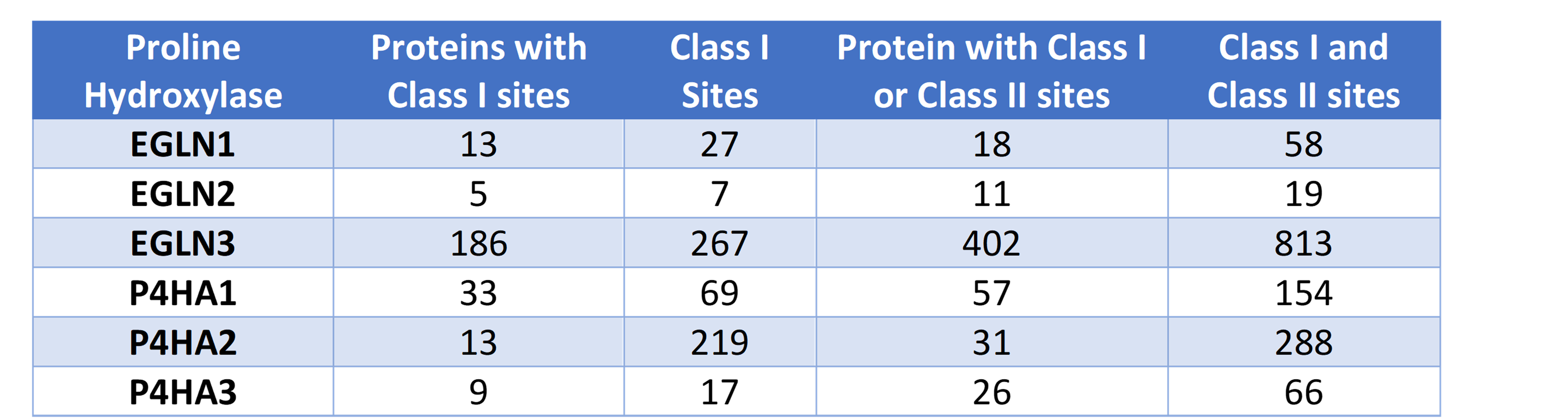

Supplement: S4 Fig — (TIF) [file pbio.3001757.s004.tif]

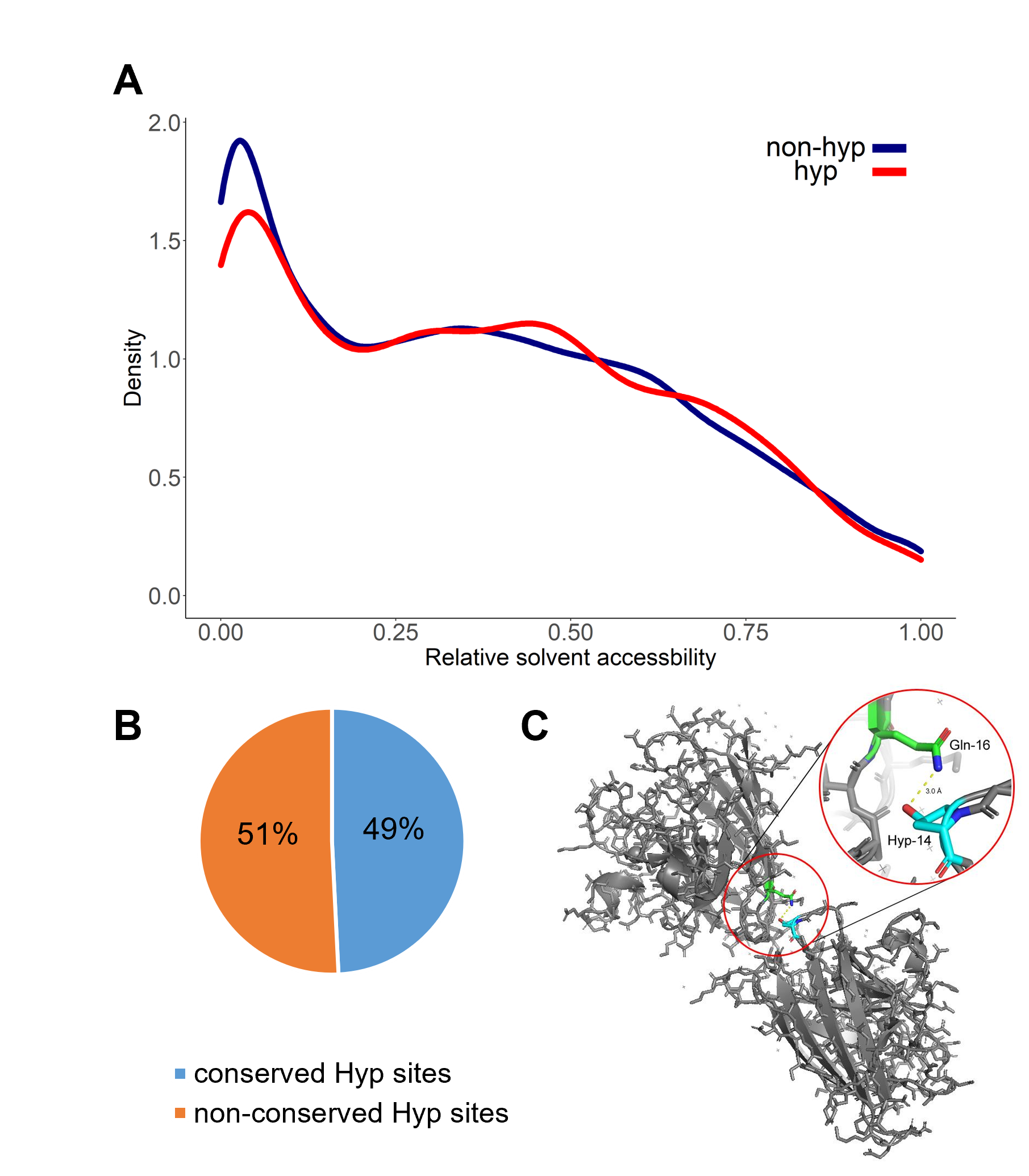

Supplement: S5 Fig — (TIF) [file pbio.3001757.s005.tif]

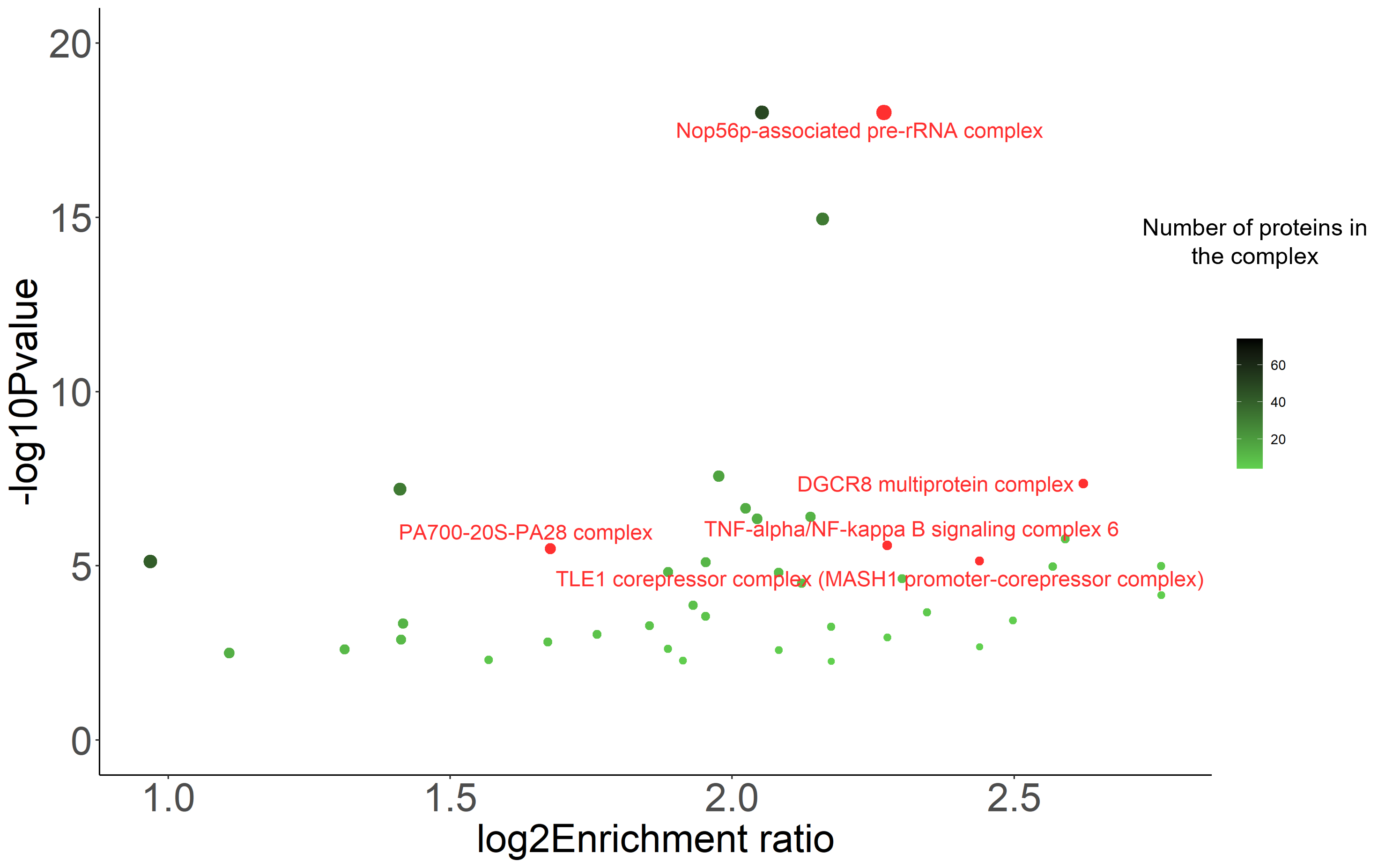

Supplement: S6 Fig — (TIF) [file pbio.3001757.s006.tif]

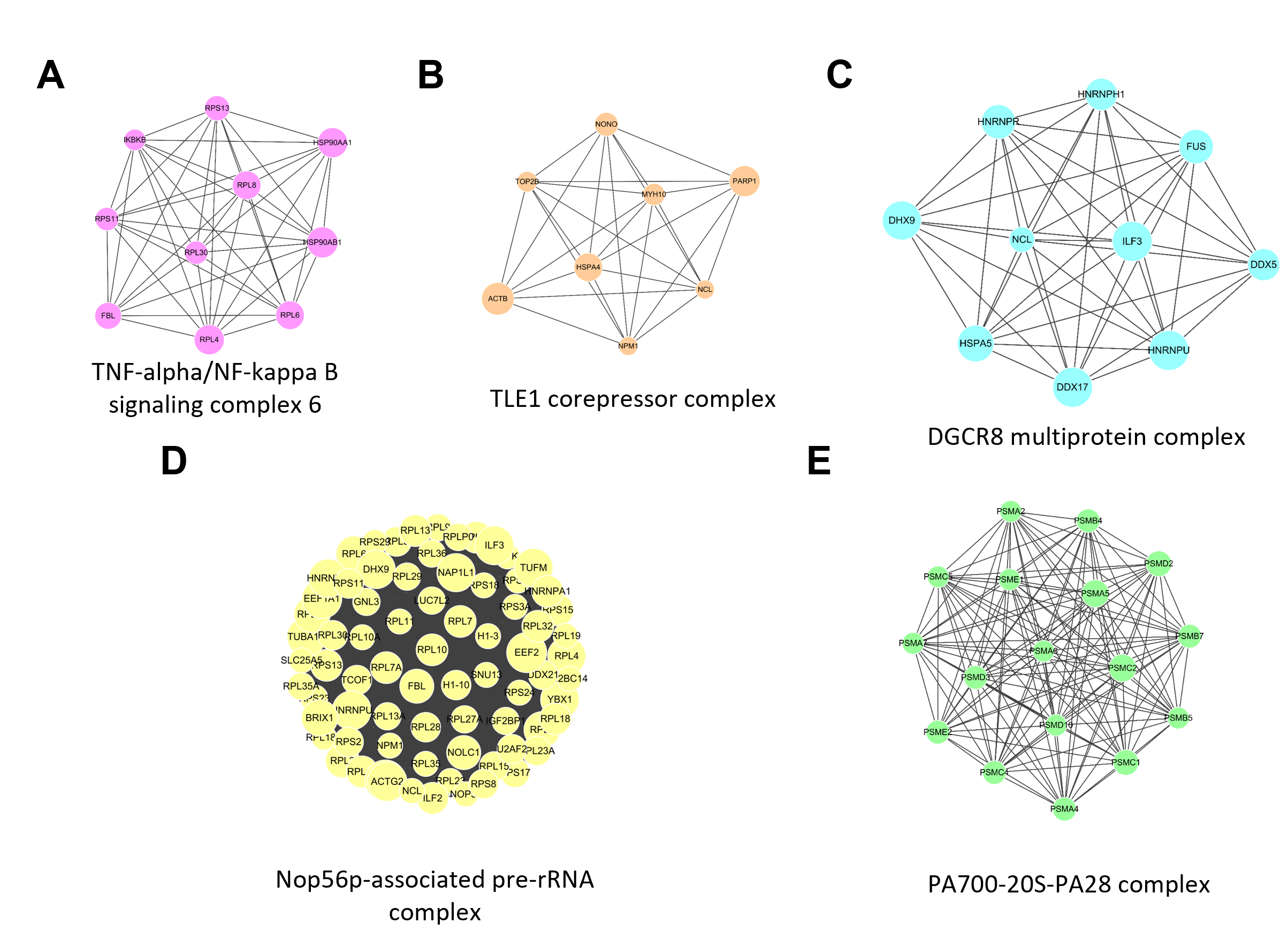

Supplement: S7 Fig — (TIF) [file pbio.3001757.s007.tif]

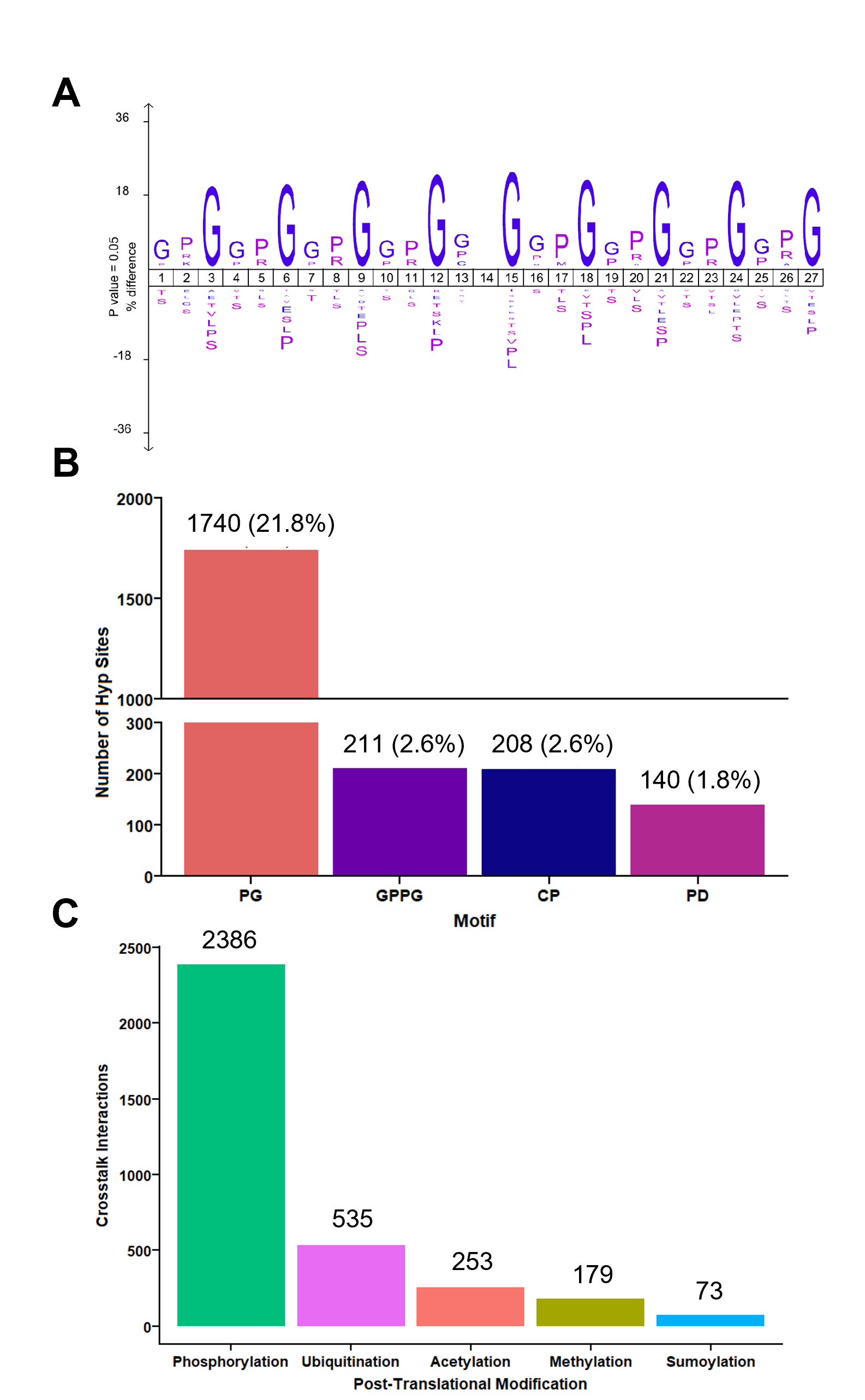

Supplement: S8 Fig — (TIF) [file pbio.3001757.s008.tif]

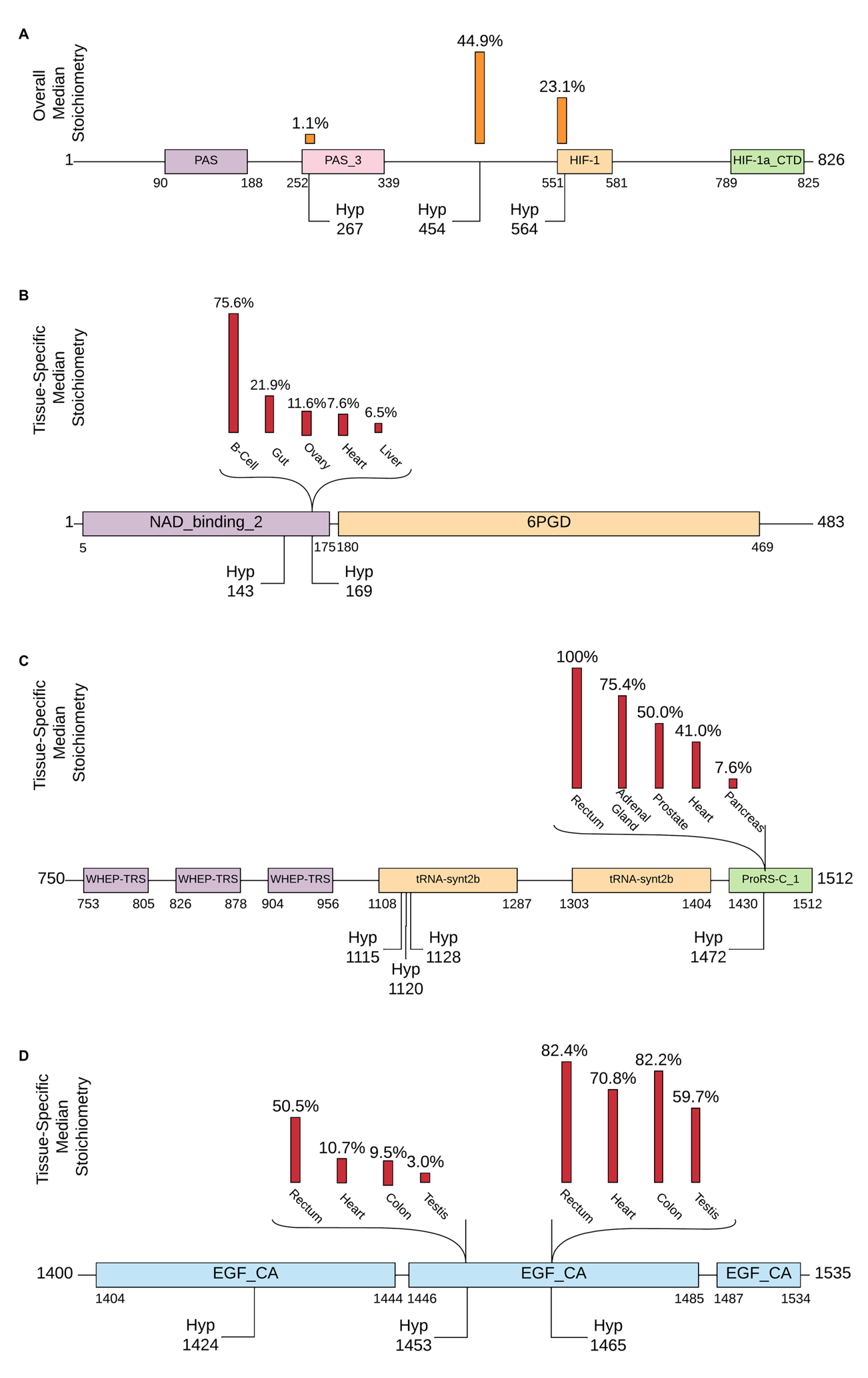

Supplement: S9 Fig — (TIF) [file pbio.3001757.s009.tif]

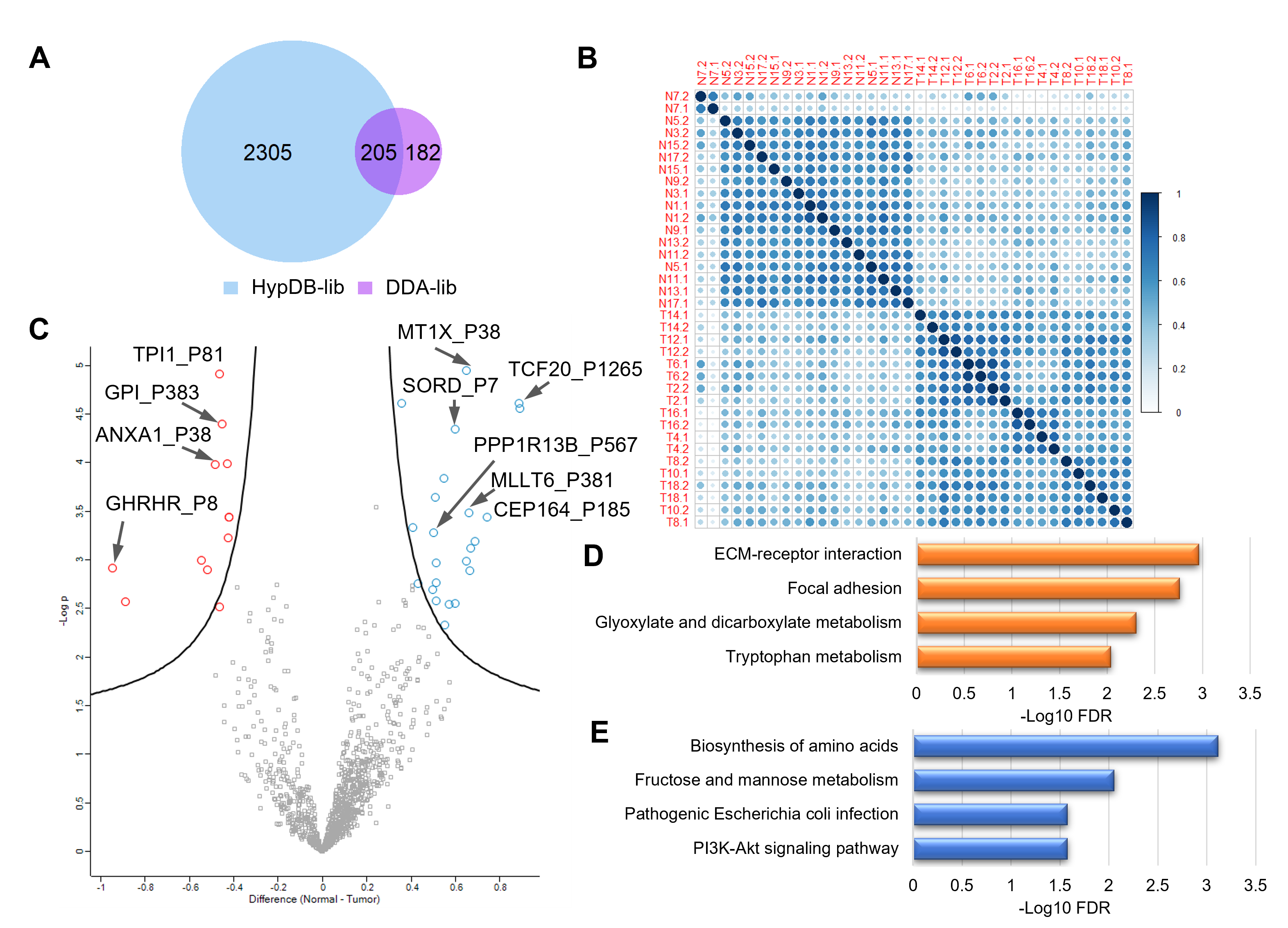

Supplement: S10 Fig — (TIF) [file pbio.3001757.s010.tif]

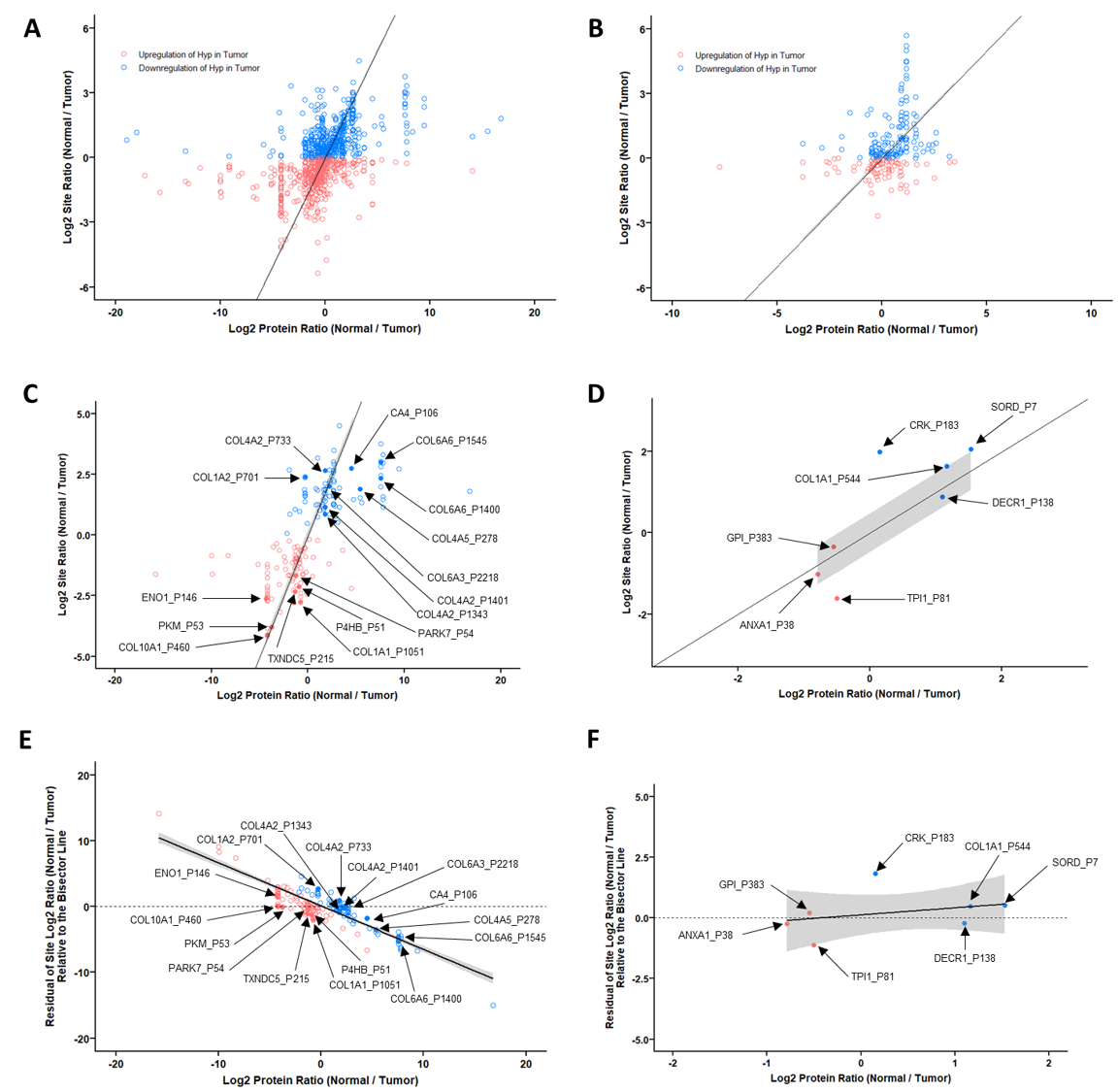

Supplement: S11 Fig — (TIF) [file pbio.3001757.s011.tif]
